# Improving Transcriptome Fidelity Following Synovial Tissue Disaggregation

**DOI:** 10.3389/fmed.2022.919748

**Published:** 2022-08-10

**Authors:** David L. Boyle, Edward B. Prideaux, Joshua Hillman, Wei Wang, Gary S. Firestein

**Affiliations:** ^1^Department of Medicine, University of California, San Diego, San Diego, CA, United States; ^2^Department of Chemistry and Biochemistry, University of California, San Diego, San Diego, CA, United States

**Keywords:** rheumatoid arthritis, synovia, functional genomics, transcriptome, disaggregation

## Abstract

**Objective:**

To improve the fidelity of the cellular transcriptome of disaggregated synovial tissue for applications such as single-cell RNA sequencing (scRNAseq) by modifying the disaggregation technique.

**Methods:**

Osteoarthritis (OA) and rheumatoid arthritis (RA) synovia were collected at arthroplasty. RNA was extracted from intact or disaggregated replicate pools of tissue fragments. Disaggregation was performed with either a proprietary protease, Liberase TL (Lib) as a reference method, Liberase TL with an RNA polymerase inhibitor flavopyridol (Flavo), or a cold digestion with subtilisin A (SubA). qPCR on selected markers and RNAseq were used to compare disaggregation methods using the original intact tissue as reference.

**Results:**

Disaggregated cell yield and viability were similar for all three methods with some viability improved (SubA). Candidate gene analysis showed that Lib alone dramatically increased expression of several genes involved in inflammation and immunity compared with intact tissue and was unable to differentiate RA from OA. Both alternative methods reduced the disaggregation induced changes. Unbiased analysis using bulk RNAseq and the 3 protocols confirmed the candidate gene studies and showed that disaggregation-induced changes were largely prevented. The resultant data improved the ability to distinguish RA from OA synovial transcriptomes.

**Conclusions:**

Disaggregation of connective tissues such as synovia has complex and selective effects on the transcriptome. We found that disaggregation with an RNA polymerase inhibitor or using a cold enzyme tended to limit induction of some relevant transcripts during tissue processing. The resultant data in the disaggregated transcriptome better represented the *in situ* transcriptome. The specific method chosen can be tailored to the genes of interest and the hypotheses being tested in order to optimize the fidelity of technique for applications based on cell suspensions such as sorted populations or scRNAseq.

## Introduction

Sorted cell-based assays, such as scRNA-seq, are increasingly important tools to analyze gene expression and pathogenic pathways in human disease (1). These methods are particularly applicable to peripheral blood cells, non-adherent cells in body fluids, or loosely bound cells such as splenocytes (2). However, single cell analysis in solid tissues has unique challenges. Disaggregation of solid tissue, particularly connective tissues such as synovium, requires significant processing that includes exposure to enzymes or mechanical stress to liberate free cells (3). However, the process of disaggregating solid tissue using proteases can potentially introduce artifacts due to rapid induction of genes. These artifacts could distort or confound identification of critical pathways associated with pathologic states. Therefore, optimizing enzymatic methods to release cells from solid tissue while minimizing transcriptional artifacts is a critical step to improve data quality for functional genomics.

This concern has become more apparent when analyzing data in large scale studies using enzymatically dispersed synovial tissue from patients with rheumatoid arthritis (RA) (4). Commonly used methodologies have not rigorously compared gene expression in disaggregated samples to intact tissue to assess the impact of disaggregation-related transcriptional changes. To address this, we have now applied qPCR (5) of selected genes and bulk RNA-seq to disaggregated tissue and intact non-disaggregated tissue from the same tissues. In preliminary studies, we observed mRNA transcript induction varied depending on the method used. Further studies compared the selected transcripts in intact synovium with disaggregated tissue from RA and osteoarthritis (OA) joint tissue. We noted that many genes implicated in RA pathogenesis were induced using the current enzymatic disaggregation method, potentially obfuscating critical pathogenic pathways.

Based on these data, we evaluated ways to mitigate this influence with alternative approaches. In particular, we focused on using an RNA polymerase inhibitor to limit transcription induced during disaggregation and the use of cold digestion to decrease cell metabolism during disaggregation. Our data showed that the changes induced by standard methods are substantial and that modifications to the current protocols can significantly limit this artifact. While none of the methods are perfect, selection of a method that is best suited to answer specific questions related to in *situ* pathogenic transcription and pathways could improve fidelity and utility of disaggregated cell data.

## Materials and Methods

### Synovial Tissue

Synovial tissue was obtained from patients with OA and RA at the time of total joint replacement or synovectomy, as previously described. The diagnosis of RA conformed to ACR/EULAR 2010 criteria for RA (6). OA was confirmed by review of the medical record and the surgeon performing arthroplasty. The goal of our cohort was to collect samples reflecting RA and OA arthroplasty cases and evaluate the influence of disaggregation. Tissue was processed immediately after arthroplasty to emulate synovial biopsy procedures and insure appropriate sampling. Luminal synovium was identified and circa 100 mg tissue fragments were dissected, pooled into random sets of circa 8 tissue fragments (20 ± 6 mg each), fragment samples and preserved in Cryostore-10 according to the manufacturer's instructions (7). The study was approved by the Institutional Review Board of UC San Diego, and informed consent was obtained from all participants.

### Synovial Tissue Disaggregation Protocols

Two proteinase-based methods based on current Liberase TL (Lib) (Roche) methods (7) were evaluated and compared to Lib alone: (1) Bacillus licheniformis protease, Subtilisin A (Sub A) (Sigma-Aldrich), which is active at 4°C, and (2) Lib in the presence of the RNA polymerase inhibitor flavopiridol (Flavo) to prevent new transcription during disaggregation. In each case arthroplasty samples were dissected into a pool of individual fragments approximately the same size as current percutaneous biopsy samples (7). About 8 fragments were randomly selected from the pool for each disaggregation condition to decrease sampling error as previously described (5).

#### Liberase TL Protocols

Synovial tissue samples were briefly thawed 37°C, washed in RPMI, weighed, and incubated in 100x tissue mass of 100 mg/ml Liberase TL, 5 mM MgCl, 800U Dnase I, with/without 2 μM flavodiridol (Sigma-Aldrich) in RPMI at 37°C for 15 min on laboratory rotator 15 min. Disaggregated cells were filtered through a 70 μM mesh filter and washed with 1%BSA /PBS. Cells were then centrifuged pelleted at 350rcf for 10 min and resuspended in 1% BSA/PBS. They were counted and viability assessed using trypan blue dye exclusion on a hemocytometer. Cells were pelleted by microcentrifugation and preserved at −80°C for total RNA analysis (6).

#### Subtilisin A Protocol

Synovial tissue samples were briefly thawed 37°C, washed in 4°C RPMI, weighed, and incubated in 40x tissue mass of 3 mg/ml Sub A, 5 mM MgCl, 800U Dnase I at 4C on a laboratory rotator (8). Disaggregated cells were collected and preserved as for Lib but with buffers are at 4°C.

### RNA Extraction

Cell pellets were extracted with RNAstat60 1E6 cells/ml according to the manufacturer's instructions. Non-disaggregated tissue samples were briefly thawed at 37C, washed in RPMI, weighed, and extracted with RNAstat 60. For PCR, 500 ng RNA was used for first-strand cDNA synthesis (Life Technologies, NY). RNA was resuspended in TE buffer for RNA-seq.

### Quantitative Real Time PCR (qPCR)

qPCR was performed using Taqman gene expression assay primer/probe sets and GeneAmp 7,300 system (Applied Biosystems, Foster City, Canada). Three separate experiments with a total of 10 OA and 5 RA were performed. The standard curve method of PCR was performed to control for cell number differences among samples and data are reported as a ratio of Relative Expression Units (REU) between disaggregated and intact tissues (6). These assays focused on MMP1, MMP3, IL-8, MIP1α, MCP1, CXCL10, COX2, PDGFß, TNF, IL-1b and IL-6.

### Statistical Methods for QPCR Transcript Analysis

Median PCR results are expressed as the ratio between control (intact tissue) and treatment (Disaggregation method) expression levels. Using one-sample Wilcoxon signed-rank sum test with μ = 1, to identify differences between methods and intact. To account for multiple comparisons, Bonferroni method was used by adjusting the *p*-values for each biomarker based on number of comparison pairs (intact vs. Sub A, intact vs. Liberase, intact vs. Lib + Flavo). Bonferroni method-corrected Mann-Whitney U-test was applied to compare RA and OA markers after different treatments. Statistical analysis was performed by the UCSD Altman Clinical and Translational Institute Biostatistics Core.

### RNA-Seq Experiments

Sequencing of RNA libraries was performed with a NovaSeq 6,000. FASTQ files from each sample were aligned using STAR v2.7.0a (9) to the GRCh38 human reference genome downloaded from UCSC (9, 10). Quantification was performed using HTSeq v0.11.2 (11). Counts were normalized for library sequencing depth and gene length using geTMM methodology (12) in R v4.0.2. Genes in the top 50% based on median geTMM expression values across all samples were used for downstream analysis. Quality control metrics for FASTQ files were established using FastQC. Other quality control analysis was performed using MultiQC (13). Determination of reads aligning to mitochondrially-encoded genes and rRNA regions was performed with custom scripts written in R. Resulting QC metrics and analysis can be found in Supplementary Figures 1–4. Elevated levels of mitochondrial reads were observed, indicating potential cell loss under disaggregation protocols. All samples were retained for analysis.

### Pathway Enrichment Analysis

Pathway enrichment analysis was performed using Reactome Pathway Database (14) using the ReactomePA package in R. Differentially expressed genes between RA and OA for each protocol were selected based on having RA/OA ≥ 1.5 using median values for each group. These genes were then submitted for analysis by Reactome for pathway enrichment. Pathways for each protocol were selected based on having Adjusted *P*-Value ≤ 0.01. *P*-values were adjusted based on Benjamini-Hochberg procedure.

## Results

### Disaggregated Cell Viability and Yield

Following disaggregation of pooled synovial tissue fragments, cells were counted on a hemocytometer by trypan blue exclusion and reported as cells/mg of tissue. Viability was evaluated by determining the percentage of cells that excluded trypan blue dye. Results from disaggregation by the three methods for RA and OA are shown in Table 1. As expected, RA yielded more cells per mg than OA. Cell yields were not significantly different for the 3 disaggregation methods, although there was a trend for a difference between Lib vs. SubA for OA (*p* = 0.06). Viability was greater for SubA compared to Lib (OA *p* = 0.013; RA *p* = 0.023) or Lib + Flavo (OA *p* = 0.02; RA *p* = 0.0002). Lib and Lib+Flavo were not significantly different from each other.

**Table 1:** Cell yield and viability for RA and OA after disaggregations.

	**Lib**			**Lib+Flavo**			**Sub A**		
	**Yield (cells/mg)**	**Viability (%)**	***N***	**Yield (cells/mg)**	**Viability (%)**	***N***	**Yield (cells/mg)**	**Viability (%)**	***N***
RA	4941 ± 4232	78 ± 6	7	5698 ± 5823	79 ± 6	7	6341 ± 5170	93 ± 3.6	7
OA	803 ± 697	75 ± 13	8	1290 ± 316	79 ± 3	10	2060 ± 1750	90 ± 13	18

*Disaggregation of RA tissue yielded more cells than OA tissue but was not significantly different among techniques. Sub A improved viability of disaggregated cells compared to Lib (OA p = 0.013; RA p = 0.023)*.

### QPCR of Candidate Genes in Intact Synovial Tissue

**Figure 1:**
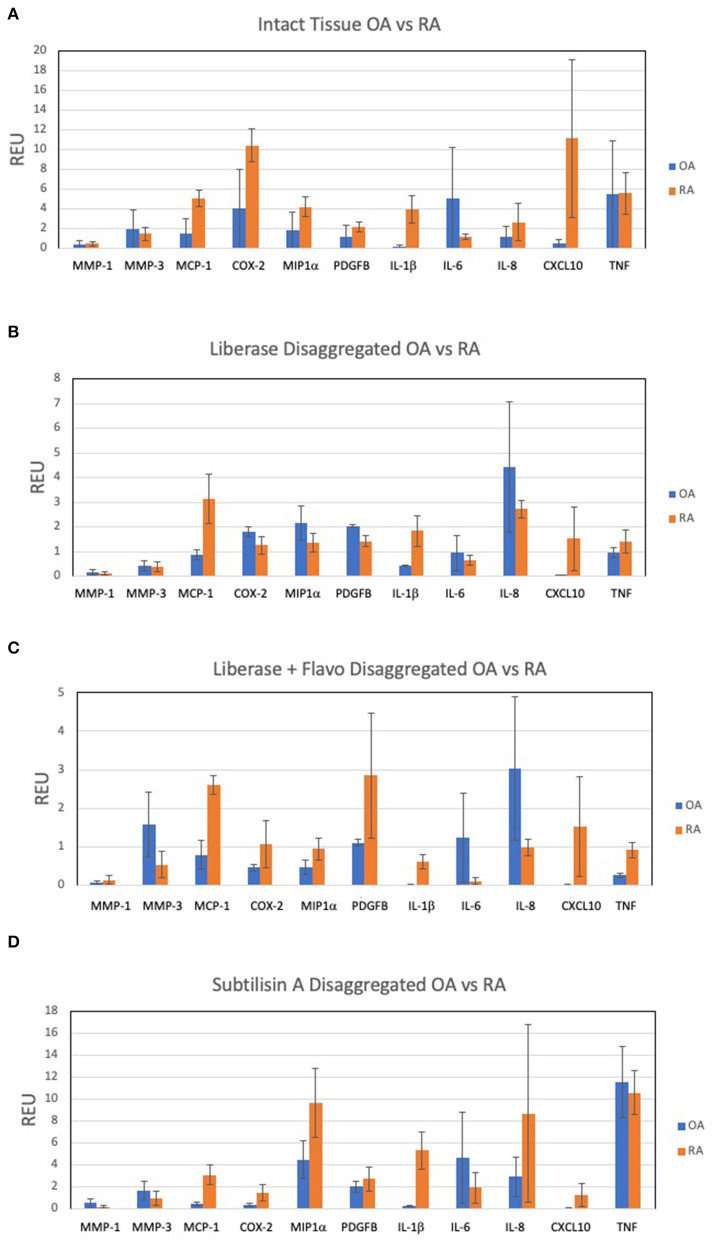
Expression of select 11 transcripts in intact synovia. Selected transcripts were measured by qPCR in replicate pools of intact OA (*n* = 10) and RA (*n* = 5) synovial tissues from 3 experiments and reported as Relative Expression Units (REU) as previously described (5). The heterogeneity of expression among tissues, markers and disease are notable. **(A)** OA and RA intact tissue, demonstrating *in situ* expression pattern. **(B)** Lib disaggregated OA and RA cells, **(C)** Lib/Flavo disaggregated OA and RA cells. **(D)** Subtilison A disaggregated synovial tissue cells.

A panel of candidate genes was used to evaluate disaggregation effects on mRNA transcripts and include MMPs, chemokines, prostanoid regulation, cytokines and growth factors. These analytes broadly represented genes implicated in synovitis and are derived from multiple different cell lineages. We first established an expression reference for disaggregated tissues by performing qPCR on extracts of intact tissue. Figure 1A shows results for the transcripts of intact RA and OA tissue and demonstrates heterogeneity, with expected overlap between OA (*n* = 10) and RA (*n* = 5). However, IL-1ß, MCP1 and CXCL10 significantly distinguished OA and RA cohorts (corrected *p* = 0.007, 0.029, and 0.015, respectively) in our patient sample.

### QPCR of Candidate Genes in Disaggregated Synovial Tissues

Expression of the gene panel was then measured in paired (to Figure 1A) disaggregated cells from OA and RA tissues by qPCR (5). Lib disaggregation (Figure 1B) increased the relative expression of IL-1ß by 4.44-fold (2.45–8.07) *p* = 0.003; IL-6 by 3.11-fold (1.39–7.91) *p* = 0.009; and IL-8. by 3.77-fold (2.06–13.95) *p* = 0.007 in OA. Adding the RNA polymerase inhibitor flavopiridol to Liberase during disaggregation reduced the effect of Liberase alone (Figure 1C). These data suggest that active transcription contributes to the effect of Liberase by inducing rapid transcription during the incubation. As an alternative to inhibiting RNA polymerase, the disaggregation was also performed at 4°C with subtilisin A. SubA tissue disaggregation decreased disaggregation effect overall for the candidate genes with the exception of TNF in OA [2.08-fold (1.28–3.12) *p* = 0.029] (Figure 1D).

### Comparison of RA vs. OA Candidate Gene Expression Following Disaggregation Protocols

**Figure 2:**
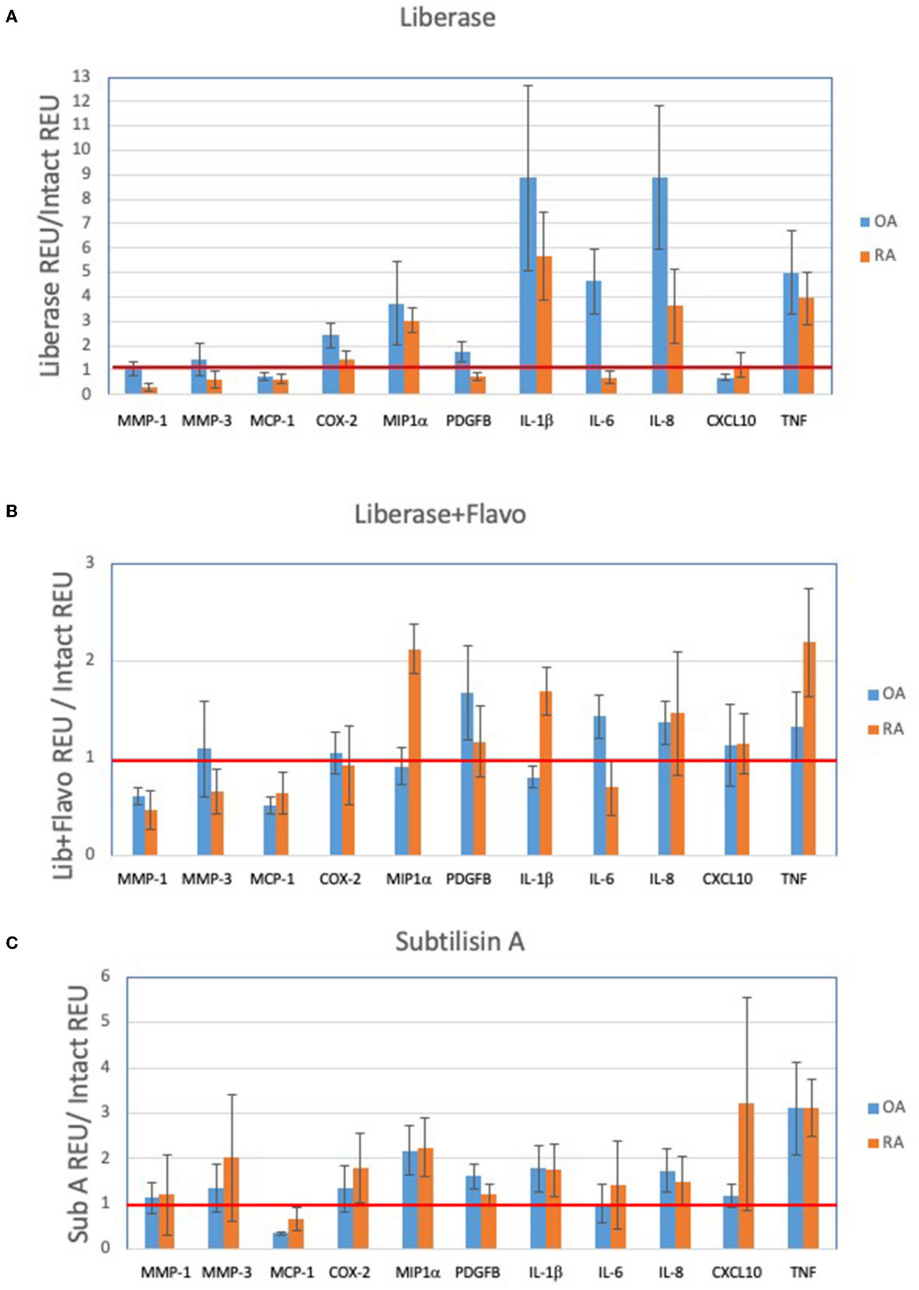
Effect of disaggregation on select 11 transcripts. Expression of candidate genes in disaggregated tissue is normalized to expression in parent (intact) tissue. **(A)** Lib, **(B)** Lib+Flavo, and **(C)** SubA. Line at 1.0 indicates identical disaggregated and intact expression, above 1.0 indicates induction or enrichment and below 1.0 indicates lower gene expression after disaggregation. Normalization illustrates the differential effects of disaggregation on individual transcripts. Note scale differential effects of disaggregation.

In order to assess the effect of disaggregation methods on OA and RA tissue cells as a changes in relative expression, the data in Figure 1 is displayed in Figures 2A–C as a ratio of disaggregated tissue cells to intact tissue, where a value of “1” indicates identical expression levels between the two. Lib disaggregation (Figure 2A) increased the relative expression of IL-1ß by 4.44-fold (2.45–8.07) *p* = 0.003; IL-6 by 3.11-fold (1.39–7.91) *p* = 0.009; and IL-8. by 3.77-fold (2.06–13.95) *p* = 0.007 in OA. Adding the RNA polymerase inhibitor flavopiridol to Liberase during disaggregation reduced the effect of Liberase alone (Figure 2B). These data suggest that active transcription contributes to the effect of Liberase by inducing rapid transcription during the incubation. As an alternative to inhibiting RNA polymerase, the disaggregation was also performed at 4°C with subtilisin A. SubA tissue disaggregation decreased disaggregation effect overall for the candidate genes with the exception of TNF in OA [2.08-fold (1.28–3.12) *p* = 0.029] (Figure 2C).

### Discriminating OA and RA Synovial Tissue Cells Following Disaggregation

We then explored the ability of the 3 protocols to discriminate between OA and RA tissues following disaggregation for the selected transcription panel (Figure 2). After disaggregation with Lib alone, none of the markers were significantly different between the two diseases using corrected *p* values (Figure 2A). However, there was a trend for IL-1ß with an uncorrected p value of 0.036. With the addition of the RNA polymerase inhibitor, flavopiridol, CXCL10, IL-1ß and MCP1 were significant (uncorrected *p* = 0.028, *p* = 0.036, *p* = 0.036, respectively) (Figure 2B). Of interest, SubA disaggregation of OA and RA showed differences in those three markers even with corrected *p* values (IL-1ß *p* = 0.007, MCP1 *p* = 0.007, CXCL10 *p* = 0.015) (Figure 2C).

### RNA-Seq Analysis of Gene Expression for Intact vs. Disaggregation Protocols

We then examined the effect of the different disaggregation protocols on broader expression patterns using unbiased RNA-seq. We performed bulk RNA-seq on 51 samples separated into 4 groups based on the protocol used to prepare the library for RNA-seq. Pools of tissue fragments from RA and OA synovia were processed for direct RNA extraction (intact tissue) or disaggregation by Lib, Lib+Flavo, or SubA from the same donors. After preparing the RNA, each sample was analyzed by RNA-seq.

**Figure 3:**
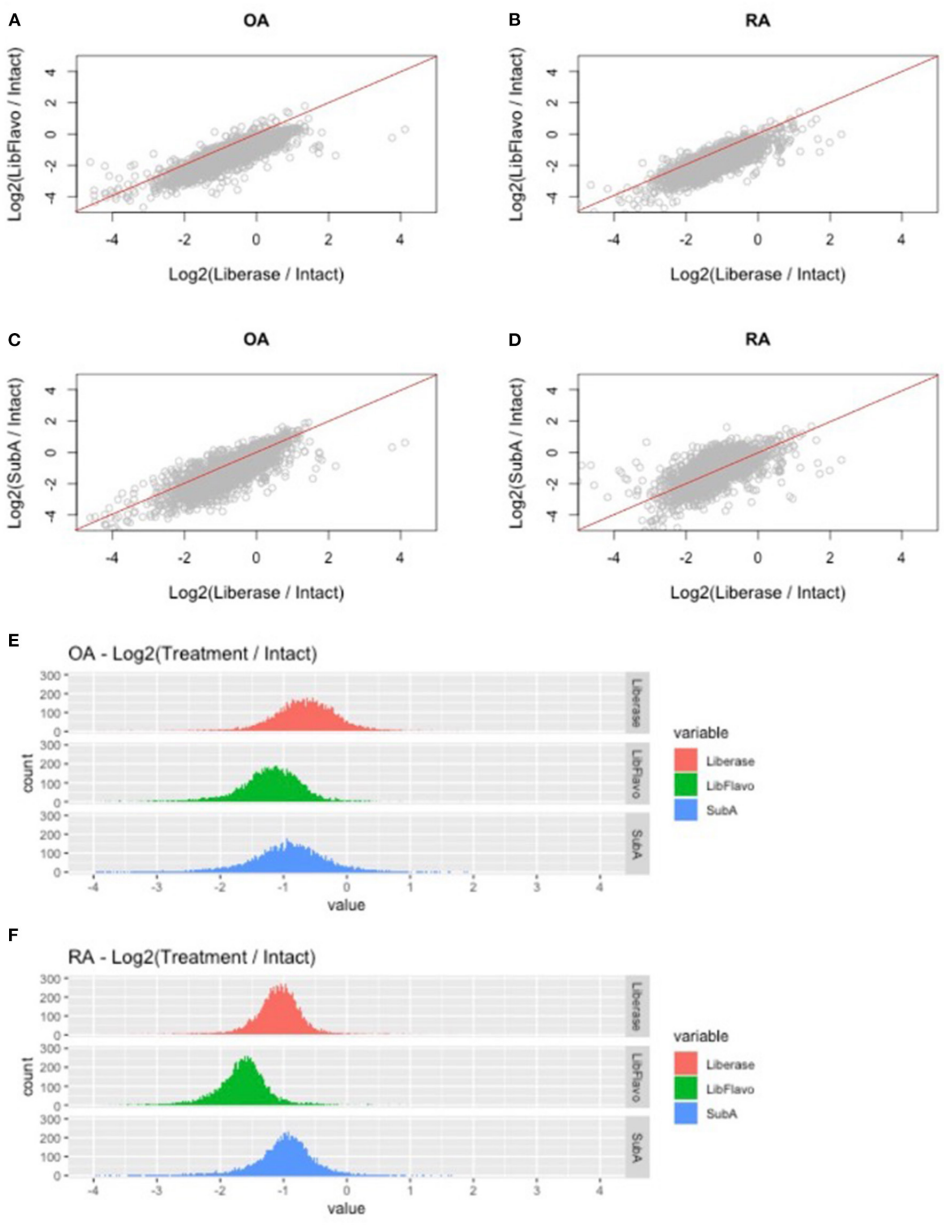
RNAseq fold change comparison of gene expression using different disaggregation protocols. **(A–D)** Relative changes in each gene for the 3 protocols compared to intact are shown for the top 50% of expressed genes. Genes that are represented below the line are induced more in Lib compared with Lib+Flavo or SubA. **(E,F)** Distribution of gene expression changes in each protocol for RA and OA. Histograms are colored by disaggregation protocol. A log2 scale is used on the x-axis, so “zero” represents identical expression compared with intact. Points to the right represent induced genes.

After processing and normalization of the data, we compared the expression levels of genes in each disaggregation protocol compared to its expression level in intact tissue (listed in Supplementary Table 1). For both RA and OA, and as noted in our qPCR studies, we observed protocol-specific changes in each disaggregation method relative to intact tissue (Figures 3A–D). The greatest changes through induction of gene expression occurred with Lib for RA and OA, which is visualized by a downward shift in the distribution of gene induction. In this analysis, Lib + Flavo and SubA were superior to Lib for RA and Lib + Flavo was superior for OA.

To understand the distribution and drivers of RNA-seq differences with each protocol, we developed histograms of expression level changes of each gene for the 3 disaggregation protocol compared to intact control tissue (Figures 3E,F). Because all libraries were normalized to equivalent sequencing depths while analyzing reads from all genes, samples with a greater percentage of mitochondrial reads would lower levels of remaining reads. Mean percentage of reads mapping to mitochondrial regions were 5.9% for OA and 7.4% for RA intact tissue. Disaggregation increased overall mitochondrial reads to 16% for lib, 24% for Lib + flavo, and 17% for Sub A.

In addition to this shift, the variance of the distribution in each protocol and disease is not uniform. For example, OA tissues have a greater variance than RA as visualized by a broader distribution in the histogram. Moreover, the variance in each protocol is different in each disease with Lib and SubA showing higher levels than Lib+Flavo, with variances of 0.34, 0.19; 0.29, 0.23, and 0.48, 0.32 for OA, RA in Lib, Lib+Flavo, and SubA, respectively.

### Comparison of RA vs. OA Unbiased Gene Expression Using Disaggregation Protocols

**Figure 4:**
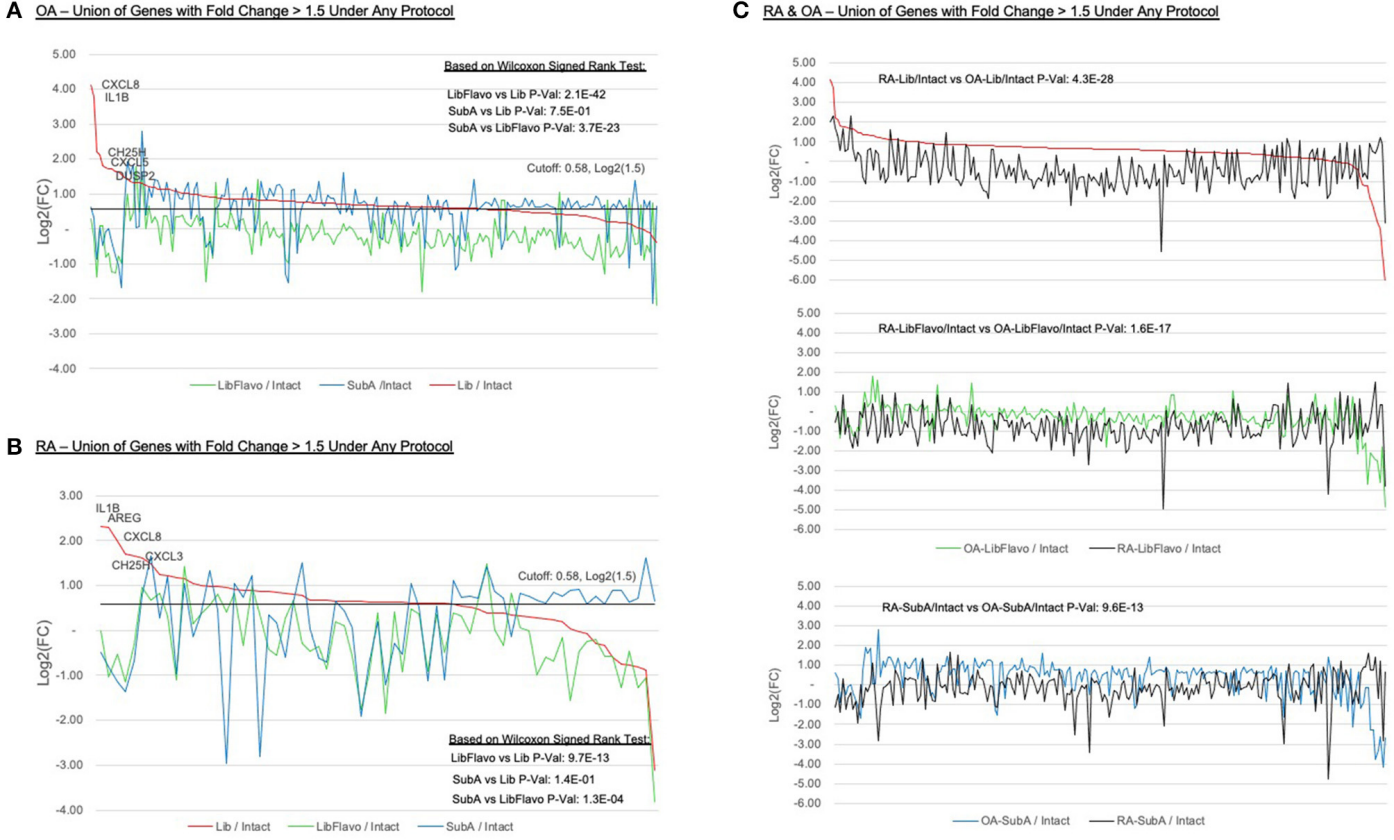
Expression ratios of induced genes under treatment protocols. Relative expression of all genes with >1.5-fold induction in each protocol relative to intact in OA **(A)** and RA **(B)**. Y-axis indicates log2(Protocol/Intact) value for each selected gene. “Zero” on the y-axis indicates no change in expression between protocol and intact. Genes are ordered by fold induction observed in Lib compared to Intact. Lines colors represent the disaggregation protocols. *P*-values calculated using Wilcoxon signed-rank method. **(C)** Inducement levels of all genes with >1.5-fold induction in each protocol relative to intact, displaying both OA (colored line) and RA (black line) for each protocol. Genes are ordered by fold induction observed in Lib compared to intact. *P*-values calculated using Wilcoxon signed-rank method. A greater difference in the RA and OA lines in each protocol indicates a greater difference in induction levels by that protocol for OA and RA.

We were particularly interested in which genes were most affected in each protocol relative to intact control tissues. For both RA and OA, we identified the genes with >1.5-fold increase due to disaggregation with each protocol relative to intact and plotted them in Figures 4A,B (see Supplementary Table 2 for list). In RA and OA, we observed significantly less gene induction in Lib+Flavo compared to Lib or SubA (RA *p*-values = 19.7x10^−13^ and 1.3x10^−4^, respectively; OA *p*-values = 2.1x10^−42^ and 3.7x10^−23^, respectively). The differences between induction patterns of Lib and SubA were not statistically significant. The genes that were increased by Lib the most correlated with the qPCR data. For example, *CXCL8, IL1B*, and *CH25H* are each present in the top 5 with Lib but are induced at lower levels in both Lib+Flavo and SubA. To compare gene induction levels between OA and RA, Figure 4C shows all genes with >1.5-fold induction in each protocol relative to intact, displaying both OA (colored line) and RA (black line) for each protocol. Genes are ordered by fold induction observed in Lib compared to Intact. *P*-values calculated using Wilcoxon signedrank method. A greater difference in the RA and OA lines in each protocol indicates a greater difference in induction levels by that protocol for OA and RA.

### Comparison of RA vs. OA Unbiased Pathways Using Disaggregation Protocols

We then investigated whether these effects were reflected in pathway enrichment for each protocol using the differentially expressed genes between RA and OA (Table 2). Each disaggregation protocol identified a broad set of pathways enriched in RA that have been implicated in pathogenesis; however, notable differences were observed. No method of disaggregation matched the analysis of intact tissue samples, which identified 21 significantly enriched pathways. We then identified the pathways that each protocol missed compared to intact tissue (Table 2). All methods missed some key immune-related pathways. However, Lib missed the most pathways compared to Lib+Flavo and SubA. Key pathways missed by Lib, but not by Lib+Flavo or SubA include *Interleukin-10 signaling* and *Signaling by NOTCH3*.

**Table 2:** Differential pathways lost or enriched by each disaggregation protocol relative to intact tissue.

**Pathway description**	**Intact tissue**	**Sub A**	**Liberase + Flavo**	**Liberase**
Interleukin-4 and Interleukin-13 signaling	6.4E−08	2.5E−06		
Assembly of collagen fibrils and other multimeric structures	9.8E−05	6.0E−03		
Interleukin-10 signaling	3.4E−08	2.7E−05	1.4E−03	
Signaling by Interleukins	1.1E−06	3.9E−04	2.4E−03	
Signaling by NOTCH3	3.5E−03	1.5E−03	9.7E−03	
Extracellular matrix organization	6.4E−08	5.6E−10	3.8E−06	3.9E−06
Chemokine receptors bind chemokines	1.5E−06	2.7E−05	1.0E−04	3.3E−06
Immunoregulatory interactions between a Lymphoid and a non-Lymphoid cell	6.4E−08	1.2E−04	2.4E−05	5.5E−08
Integrin cell surface interactions	4.8E−07	1.3E−04	4.3E−05	3.9E−05
GPCR ligand binding	1.2E−03	4.5E−04	3.9E−04	1.3E−04
Class A/1 (Rhodopsin-like receptors)	5.5E−05	7.9E−04	3.0E−05	1.7E−05
Peptide ligand-binding receptors	1.3E−04	7.2E−03	8.0E−06	3.1E−05
G alpha (i) signaling events	1.4E−03	4.5E−03	3.0E−03	1.7E−04
Binding and Uptake of Ligands by Scavenger Receptors	2.8E−05			
Collagen degradation	2.8E−05			
Degradation of the extracellular matrix	5.7E−05			
Collagen chain trimerization	2.0E−03			
RAF-independent MAPK1/3 activation	2.1E−03			
Collagen formation	3.0E−03			
NOTCH3 Intracellular Domain Regulates Transcription	3.1E−03			
Interleukin-6 signaling	8.4E−03			
Scavenging of heme from plasma		9.1E−03		1.4E−03
Elastic fiber formation		2.4E−07		
Molecules associated with elastic fibers		3.1E−04		
Signaling by NOTCH1		4.5E−03		
Erythrocytes take up carbon dioxide and release oxygen			3.0E−03	
O2/CO2 exchange in erythrocytes			3.0E−03	
Syndecan interactions			4.7E−03	
Triglyceride metabolism				8.6E−03

## Discussion

Techniques such as scRNA-seq or sorted-cell transcriptional analysis offer unprecedented resolution and sensitivity of individual cell transcriptomes (15). scRNA-seq is particularly applicable to liquid tissues such as blood or to tissues where cells are loosely bound to matrix such as spleen or bone marrow (15). However, tissues with a major connective tissue component require disaggregated by various approaches that include combinations mechanical and enzymatic methods to release cells that adhere to the extracellular matrix (7, 16, 17).

Disaggregation methods are typically optimized for cell yield and viability, but the impact on the transcriptome is typically not evaluated or analyzed. Using current methods, the resultant transcriptome can define cell lineages and subsets in a dispersed tissue (7). However, rapid induction of some critical genes during disaggregation might obfuscate information required to determine *in situ* gene expression and pathways.

Our work to study synovial functional transcriptomics led us to observe that many genes critical to immune processes and inflammation can be affected by standard enzymatic disaggregation methods. Current recombinant enzymes cocktails such as the Liberase family improved upon earlier crude protease extracts. However, we discovered that the tissue processing induces transcription of many genes. We therefore studied two alternatives to mitigate this concern, namely an RNA polymerase inhibitor such as flavopiridol to limit de novo transcription or Subtilisin A to disaggregate the tissue at lower temperature by lowering tissue metabolic activity. Cell yields were similar to standard methods with some improvement of viability with the cold disaggregation method.

Time, temperature, enzymes, inhibitors and the size of tissue fragments are variables that are considered during method development. 15 min for digestion is a minimum in our hands for reproducible handling and tissue fragments were small as the technique was designed to evaluate needle biopsies. Given the heterogeneity of synovial tissue within a single joint, multiple individual tissue “biopsies” from arthroplasty synovium were pooled and randomly allocated to minimize the impact of sampling error (6, 18). A unique aspect of this study was our comparison of disaggregated cells to intact parent tissue. This demanding analysis integrates cell loss and damage as well as stimulation that occurs during processing.

We initially selected a set of candidate genes that reflect diverse regulatory mechanisms and cellular origins for measurement by qPCR in intact and disaggregated tissue. IL-1ß, CXCL10 and MCP1 expression could discriminate between the RA and OA tissues (19) in intact tissue, confirming data from studies showing that cytokine and chemokine expression is higher in RA than OA synovium (20–23). Of interest, some genes like MMPs were not affected by disaggregation, possibly because the study was underpowered or the variability was too great for that analysis.

The most striking observation was the dramatic increase in gene expression induced by the short enzymatic digestion time for many genes implicated in immunity using a standard Lib protocol relative to intact tissue (7, 24). As a result, the ability to distinguish gene expression patterns for RA compared with OA could be lost. The addition of flavopiridol or use of subtilisin A substantially decreased disaggregation induced gene expression. Some candidate genes were not affected, but the modified methods preserved RA-OA differences for some highly induced genes like IL-1ß following disaggregation.

We then performed an unbiased analysis by comparing bulk RNA-seq of matched intact tissue and disaggregated cells from the parent tissue. The data confirmed that Lib alone had a dramatic effect on the transcriptome and induced about 600 genes, many of which are critical to RA pathogenesis. As with our candidate gene approach, the addition of Flavo or use of SubA largely mitigated this artifact and improved discrimination between RA and OA. We also noted that many RA-OA differential pathways that were lost using the Liberase protocol involved multiple immunologic processes but were restored with the alternative protocols.

It is important to note that none of the methods exactly replicate the transcriptome of intact tissue. Each protocol has a different pattern of differential expression that distinguish disaggregated samples from intact tissue. In addition, RNA quality could be adversely affected by the alternative methods and affect quantification. This could contribute to the leftward shift in the histograms in Figure 3A, although cell loss during the procedure is an alternative explanation. Nevertheless, the ability to discriminate pathways minimize disaggregation induction appeared to be optimal for Lib + Flavo, particularly for OA (Figure 3A). SubA followed by the Lib + Flavo method were optimal to distinguish RA from OA based the pathway analyses. Both of those methods were superior to Lib alone. The specific protocol used, however, can be tailored to the hypothesis that is tested and the genes of interest.

Our RNA-seq dataset should allow investigators to customize their approaches based on which genes are most germane, although the methods still need to be confirmed using scRNA-seq methods. In the future, spatial functional transcriptomics techniques might provide improved resolution and sequencing depth to determine in *situ* gene expression and ultimately prove to be superior to enzymatic digestion. In the interim, application of the methods that we describe could improve the fidelity of RNA-seq data for identifying pathogenic genes in diseases like RA.

## Data Availability Statement

The data/analyses presented in the current publication have been deposited in and are available from the dbGaP database under dbGaP accession #phs002991.v1.

## Ethics Statement

The studies involving human participants were reviewed and approved by the UCSD Institutional Review Board (protocol # 140175). The patients/participants provided their written informed consent to participate in this study.

## Author Contributions

DB and GF designed studies. EP performed analysis of RNAseq data. WW interpreted RNAseq data. JH performed synovial disaggregations. All authors contributed to the article and approved the submitted version.

## Funding

This work was supported in part by the Allen Institute of Immunology, the National Institutes of Health RA/SLE Accelerated Medicines Program 5UH2AR067681, Grants UL1TR001442 and 5R01AR071321. Publication includes data generated at the UC San Diego IGM Genomics Center utilizing an Illumina NovaSeq 6000 that was purchased with funding from a National Institutes of Health SIG grant (#S10 OD026929).

## Conflict of Interest

The authors declare that the research was conducted in the absence of any commercial or financial relationships that could be construed as a potential conflict of interest.

## Publisher's Note

All claims expressed in this article are solely those of the authors and do not necessarily represent those of their affiliated organizations, or those of the publisher, the editors and the reviewers. Any product that may be evaluated in this article, or claim that may be made by its manufacturer, is not guaranteed or endorsed by the publisher.
