# Supplementary material for: Improving Transcriptome Fidelity Following Synovial Tissue Disaggregation
Source: Front Med (Lausanne). 2022 Aug 10;9:919748. doi: 10.3389/fmed.2022.919748 (PMC9400013; doi:10.3389/fmed.2022.919748)

**Supplementary Materials**

**Supplementary Table 1: Genes where Fold Change > 1.5 between Condition and Intact for any Condition**

|  | **Fold Change** | | | | | |
| --- | --- | --- | --- | --- | --- | --- |
|  | **OA** | | | **RA** | | |
|  | **Lib / Intact** | **LibFlavo / Intact** | **SubA / Intact** | **Lib / Intact** | **LibFlavo / Intact** | **SubA / Intact** |
| CXCL8 | 4.12 | 0.30 | 0.62 | 1.99 | -0.54 | -1.15 |
| IL1B | 3.77 | -0.24 | 0.32 | 2.32 | -0.01 | -0.48 |
| CH25H | 2.20 | -1.37 | -0.88 | 1.71 | -1.14 | -1.37 |
| CXCL5 | 2.13 | 0.08 | 0.04 | 1.23 | 0.82 | 0.28 |
| DUSP2 | 1.81 | 0.10 | -0.48 | 0.57 | -1.17 | -1.24 |
| CXCL3 | 1.75 | -0.82 | -0.07 | 1.66 | -0.32 | -0.69 |
| PTGS2 | 1.74 | -0.71 | 0.01 | 0.52 | -1.36 | -0.38 |
| CXCL2 | 1.73 | -1.22 | -0.37 | 0.63 | -1.84 | -1.22 |
| AREG | 1.65 | -1.27 | -0.77 | 2.30 | -1.03 | -0.82 |
| ATF3 | 1.65 | -0.79 | -1.03 | 0.65 | -1.76 | -1.92 |
| AHSP | 1.56 | -0.96 | -1.67 | 0.00 | 0.01 | 0.00 |
| CD83 | 1.48 | 0.15 | 0.68 | 0.48 | -0.31 | -1.15 |
| CD300E | 1.44 | 1.00 | 1.91 | 0.24 | 0.43 | -0.16 |
| SLC16A6 | 1.35 | 0.22 | 1.60 | -0.78 | -1.24 | -0.36 |
| SLC46A2 | 1.34 | 0.46 | 1.85 | -1.14 | -0.59 | 0.06 |
| S100A12 | 1.34 | 1.79 | 0.04 | 0.57 | 0.39 | 1.10 |
| GPR183 | 1.33 | 0.50 | 0.07 | 0.67 | -0.36 | -0.62 |
| CCL7 | 1.30 | 1.60 | 2.78 | -0.44 | -1.62 | -2.83 |
| RGS1 | 1.25 | 0.02 | 0.39 | 0.58 | -1.05 | -1.23 |
| CYTIP | 1.21 | 0.65 | 1.20 | 0.35 | 0.83 | -0.13 |
| BMP2K | 1.20 | 0.17 | 0.92 | -0.94 | -1.32 | -0.10 |
| TLR8 | 1.18 | 0.35 | 1.41 | -0.31 | -0.18 | 0.08 |
| NCEH1 | 1.14 | 0.18 | 1.40 | -1.09 | -1.33 | 0.20 |
| CD28 | 1.14 | -0.45 | 1.11 | -0.79 | -1.05 | -1.04 |
| KCNA3 | 1.12 | 0.37 | 0.80 | 1.62 | 0.96 | 0.78 |
| LYZ | 1.12 | 0.38 | 1.33 | 0.39 | -0.21 | 0.46 |
| DMXL2 | 1.12 | 0.11 | 0.93 | -0.67 | -1.21 | 0.14 |
| TNFAIP3 | 1.11 | -0.63 | -0.14 | 1.17 | -1.11 | -0.97 |
| CTSS | 1.08 | 0.35 | 1.15 | -0.70 | -0.49 | 0.35 |
| RNASE2 | 1.06 | 0.34 | 0.87 | -0.31 | -0.83 | 0.23 |
| CLEC5A | 1.03 | 0.41 | 0.86 | 1.00 | 0.36 | 0.37 |
| CYBB | 1.03 | 0.20 | 1.14 | -1.04 | -0.79 | 0.31 |
| OLR1 | 1.01 | 0.11 | 1.34 | -0.75 | -0.96 | 0.25 |
| OSM | 1.01 | 0.06 | 0.28 | -0.76 | -1.74 | -0.84 |
| SUCNR1 | 1.00 | 0.23 | 1.10 | -0.79 | -1.13 | -0.52 |
| MCEMP1 | 1.00 | 0.40 | 1.34 | 0.98 | 0.57 | 1.32 |
| CLEC4E | 0.95 | 0.08 | 0.22 | -0.28 | -0.77 | -1.23 |
| ZNF460 | 0.94 | 0.30 | 0.34 | 0.27 | -0.44 | 0.46 |
| ARC | 0.93 | -1.50 | -0.52 | 0.56 | -0.71 | -0.40 |
| BCL2A1 | 0.92 | -0.10 | -0.37 | -0.08 | -0.35 | -0.80 |
| DUSP5 | 0.91 | -0.85 | -0.74 | -1.15 | -1.61 | -0.82 |
| FCN1 | 0.90 | 1.34 | 0.60 | 0.91 | 0.82 | 1.05 |
| NLRP3 | 0.88 | 0.06 | 0.65 | 0.40 | -0.26 | -0.04 |
| PIK3AP1 | 0.87 | 0.05 | 0.97 | -1.06 | -1.26 | -0.16 |
| FPR3 | 0.87 | 0.15 | 0.93 | -0.71 | -0.54 | 0.00 |
| AQP9 | 0.86 | 0.04 | 1.45 | -0.14 | -0.41 | -0.39 |
| ENHO | 0.85 | 0.50 | 0.52 | 1.48 | 0.68 | 1.67 |
| PTPRC | 0.85 | 0.27 | 0.82 | -0.28 | -0.31 | -0.26 |
| CCDC88A | 0.85 | -0.02 | 0.79 | -0.72 | -1.16 | -0.64 |
| CX3CR1 | 0.85 | 0.22 | -0.04 | 0.77 | -0.29 | 1.49 |
| EVI2B | 0.85 | 0.28 | 1.02 | -0.93 | -0.95 | -0.41 |
| CD84 | 0.84 | -0.04 | 1.20 | -0.93 | -1.22 | -0.46 |
| NOD2 | 0.84 | -0.16 | 0.66 | -1.23 | -1.14 | -0.39 |
| TCHH | 0.84 | -0.73 | 1.01 | -0.84 | -1.24 | -0.47 |
| EREG | 0.84 | 0.33 | -0.10 | 0.85 | -0.56 | 0.18 |
| MTRNR2L1 | 0.84 | 1.43 | 0.33 | 0.61 | 0.36 | 0.52 |
| P2RY1 | 0.84 | -0.46 | 1.35 | -0.68 | -1.40 | -0.25 |
| S100A9 | 0.83 | 0.09 | 0.97 | 0.20 | -0.16 | 0.77 |
| ALDH1A1 | 0.82 | 0.19 | 0.84 | -0.94 | -1.17 | 0.38 |
| SORL1 | 0.82 | -0.26 | 1.17 | -0.58 | -0.45 | 0.29 |
| MSR1 | 0.81 | 0.03 | 0.93 | -1.12 | -0.94 | -0.06 |
| STARD4 | 0.81 | -0.19 | 1.24 | -1.47 | -1.77 | -0.83 |
| CD163 | 0.81 | -0.26 | 1.27 | -1.51 | -1.87 | -0.04 |
| TLR7 | 0.81 | -0.13 | 1.13 | -1.87 | -2.11 | -0.11 |
| PMAIP1 | 0.80 | -0.86 | -1.29 | 0.60 | -0.48 | -1.10 |
| SOCS1 | 0.80 | -0.97 | -1.54 | 0.60 | -0.90 | -1.13 |
| SIGLEC10 | 0.79 | 0.18 | 1.10 | -0.82 | -0.05 | 0.10 |
| CCR5 | 0.79 | -0.09 | 1.15 | -0.36 | -0.19 | 0.16 |
| CD1E | 0.78 | 0.14 | -0.69 | 0.14 | -0.45 | -0.15 |
| ALG10 | 0.77 | -0.38 | 0.46 | -0.12 | -0.47 | 0.38 |
| TM6SF1 | 0.76 | -0.20 | 0.73 | -0.86 | -0.80 | 0.31 |
| IKZF1 | 0.76 | 0.11 | 0.80 | -0.17 | -0.47 | -0.08 |
| LIPA | 0.76 | -0.01 | 0.99 | -0.73 | -1.52 | -0.51 |
| MPEG1 | 0.75 | -0.34 | 0.77 | -0.33 | -0.58 | 0.59 |
| TRIM58 | 0.75 | -0.15 | 0.84 | -1.08 | -1.21 | 0.00 |
| ITLN1 | 0.74 | -0.07 | 1.37 | 0.38 | -0.32 | 0.71 |
| HLA-DQA1 | 0.74 | 0.25 | 0.73 | -0.20 | -0.85 | -0.74 |
| DOCK2 | 0.73 | -0.16 | 0.66 | -0.93 | -0.65 | -0.05 |
| FCGR3A | 0.73 | -0.12 | 0.96 | -0.44 | -0.57 | 0.56 |
| TLR4 | 0.73 | -0.26 | 0.88 | -0.88 | -1.07 | 0.35 |
| LILRB4 | 0.73 | -0.43 | 0.87 | -0.90 | -1.16 | 0.04 |
| ARHGAP18 | 0.72 | -0.19 | 0.61 | -1.37 | -1.51 | -0.45 |
| LYN | 0.72 | -0.11 | 0.42 | -0.99 | -0.94 | -0.26 |
| L1TD1 | 0.72 | 0.15 | 1.62 | -0.85 | -0.38 | 0.25 |
| ITGAV | 0.70 | -0.05 | 0.49 | -0.99 | -1.23 | -0.82 |
| TNFSF8 | 0.70 | 0.08 | 0.68 | -0.63 | -0.22 | 0.06 |
| REL | 0.70 | 0.00 | 0.41 | -0.06 | -0.54 | 0.19 |
| CD180 | 0.69 | -0.12 | 1.14 | -1.27 | -1.95 | 0.28 |
| OTULINL | 0.69 | -0.30 | 0.74 | -0.52 | -0.40 | 0.32 |
| RBM47 | 0.69 | -0.25 | 0.74 | -1.15 | -1.49 | -0.51 |
| TIMD4 | 0.68 | -0.48 | 0.78 | 0.31 | -0.01 | 0.75 |
| ITGAM | 0.68 | -0.26 | 0.70 | -0.65 | -0.56 | 0.28 |
| SDS | 0.68 | -0.76 | -0.10 | -0.69 | -1.71 | -1.28 |
| SRGN | 0.67 | 0.22 | 0.64 | -0.39 | -0.20 | -0.44 |
| CCR1 | 0.66 | -0.08 | 0.90 | -1.09 | -1.08 | -0.48 |
| OTUD1 | 0.66 | -0.55 | -0.24 | -0.53 | -1.08 | -0.22 |
| TNFAIP6 | 0.66 | 0.46 | -0.29 | -2.22 | -2.24 | -2.55 |
| CYP2S1 | 0.65 | -0.36 | 0.39 | -0.11 | -0.40 | -0.69 |
| SOCS6 | 0.65 | -0.41 | 0.63 | -1.44 | -1.59 | -0.28 |
| SLC4A7 | 0.65 | -0.09 | 0.14 | -0.83 | -1.15 | -0.05 |
| MNDA | 0.65 | 0.25 | 0.79 | -0.78 | -0.93 | -0.17 |
| LAPTM5 | 0.65 | 0.01 | 0.69 | -0.76 | -0.75 | 0.01 |
| CA2 | 0.65 | -0.65 | -0.50 | -1.76 | -2.72 | -3.44 |
| HPSE | 0.65 | -0.09 | 0.55 | -0.61 | -0.38 | 0.37 |
| C2 | 0.64 | -0.50 | 0.76 | -0.97 | -1.39 | 0.09 |
| IL10RA | 0.64 | -0.39 | 0.62 | -0.76 | -0.53 | 0.21 |
| TMPPE | 0.64 | -0.07 | 0.08 | -0.96 | -1.36 | -0.02 |
| SLC25A24 | 0.64 | -0.15 | 0.50 | -1.24 | -1.53 | -0.85 |
| NFE2L3 | 0.64 | 0.19 | 0.55 | -0.55 | -0.61 | -0.48 |
| SELE | 0.63 | -1.79 | 0.64 | -1.78 | -0.69 | -0.79 |
| H2AC8 | 0.63 | -0.32 | -0.55 | -0.35 | -0.35 | -1.62 |
| FFAR4 | 0.63 | -0.41 | 0.96 | -1.38 | -1.09 | -0.77 |
| SLC43A2 | 0.62 | -0.54 | 0.68 | -1.07 | -0.95 | -0.29 |
| LPAR5 | 0.62 | -0.50 | 0.49 | -1.54 | -1.61 | -0.38 |
| SNX10 | 0.62 | -0.01 | 0.82 | -1.03 | -1.53 | -0.76 |
| PHIP | 0.62 | -0.09 | 0.26 | 0.28 | -0.56 | 0.05 |
| SLC7A7 | 0.60 | -0.51 | 0.74 | -0.77 | -0.66 | 0.22 |
| GPR34 | 0.60 | -0.35 | 0.83 | -1.28 | -2.10 | -0.61 |
| LACC1 | 0.60 | -0.13 | 0.42 | -1.69 | -1.91 | -0.73 |
| SMPDL3A | 0.60 | -0.25 | 0.43 | -1.18 | -1.08 | -0.35 |
| VGLL2 | 0.59 | -0.50 | -1.18 | 0.23 | -0.68 | 0.85 |
| NAMPT | 0.59 | -0.19 | -1.03 | -0.57 | -0.94 | -0.97 |
| SKIL | 0.59 | -0.56 | 0.49 | -1.30 | -1.42 | -0.14 |
| VPS13A | 0.59 | -0.65 | -0.34 | -0.18 | -1.37 | -0.41 |
| VPS13C | 0.59 | -0.10 | 0.53 | -0.33 | -0.87 | -0.04 |
| LILRB5 | 0.59 | -0.93 | 0.74 | -1.13 | -1.24 | -0.32 |
| MPZL2 | 0.59 | -0.45 | 1.40 | -0.86 | -1.33 | 0.04 |
| MYO5A | 0.59 | -0.41 | 0.51 | -1.37 | -1.60 | -0.58 |
| CDCP1 | 0.58 | -0.14 | 0.61 | -1.03 | -0.91 | -0.21 |
| IL10 | 0.58 | -0.37 | 0.69 | -0.13 | -0.94 | 0.01 |
| PIK3CG | 0.57 | -0.20 | 0.76 | -1.22 | -1.24 | -0.10 |
| FYB1 | 0.55 | -0.28 | 0.73 | -0.87 | -0.57 | -0.43 |
| MMP3 | 0.55 | -0.81 | 0.67 | -4.56 | -4.97 | -2.10 |
| S100A8 | 0.55 | 0.28 | 0.88 | 0.33 | 0.06 | 0.83 |
| C9orf72 | 0.54 | -0.28 | 0.61 | -1.29 | -1.27 | -0.31 |
| ITGAL | 0.54 | 0.83 | -0.58 | -0.10 | -0.32 | -0.12 |
| CD8A | 0.53 | 0.83 | -0.31 | -0.40 | 0.03 | -0.78 |
| ARL11 | 0.53 | -0.34 | 0.77 | -0.77 | -0.73 | -0.02 |
| DOCK8 | 0.53 | -0.19 | 0.62 | -0.75 | -0.88 | -0.39 |
| LILRB2 | 0.52 | -0.04 | 0.67 | -0.44 | -0.43 | 0.10 |
| ME2 | 0.52 | -0.16 | 0.62 | -1.12 | -1.45 | -0.51 |
| NCKAP1L | 0.51 | -0.40 | 0.63 | -1.15 | -1.29 | -0.13 |
| CXCR4 | 0.50 | -0.57 | 0.05 | 0.63 | -0.99 | -0.75 |
| C5AR1 | 0.49 | -0.39 | 0.67 | -0.26 | -0.55 | -0.21 |
| SOWAHD | 0.49 | 0.07 | 0.87 | -0.56 | -1.10 | -0.47 |
| GPR82 | 0.49 | -0.42 | 0.48 | 0.68 | -0.47 | 0.03 |
| FMNL2 | 0.48 | -0.66 | 0.67 | -1.50 | -1.74 | -0.46 |
| FBP1 | 0.47 | -0.24 | 0.83 | -0.07 | -0.25 | 0.59 |
| ZDBF2 | 0.47 | -0.15 | 0.29 | 0.27 | -0.98 | 0.67 |
| SYK | 0.47 | -0.40 | 0.62 | -0.90 | -0.83 | 0.06 |
| ITGB2 | 0.46 | -0.03 | 0.63 | -0.29 | -0.44 | 0.14 |
| DSC2 | 0.46 | -0.19 | 0.62 | -1.60 | -1.66 | -0.67 |
| CD86 | 0.46 | 0.03 | 0.66 | -0.59 | -0.55 | -0.23 |
| NCF1 | 0.45 | -0.56 | 0.57 | 0.53 | -0.07 | 0.76 |
| CD300LF | 0.45 | -0.17 | 0.71 | -0.40 | -0.48 | 0.05 |
| FGR | 0.44 | -0.01 | 0.65 | -0.28 | -0.25 | -0.10 |
| FPR1 | 0.43 | -0.09 | 0.62 | -0.75 | -0.57 | -0.13 |
| VSIG4 | 0.43 | -0.44 | 0.51 | -0.76 | -0.46 | 0.63 |
| IER5 | 0.43 | -0.79 | -0.20 | 0.62 | -0.93 | -0.53 |
| ADA2 | 0.43 | -0.59 | 0.61 | -1.11 | -1.31 | -0.13 |
| GNLY | 0.42 | 1.05 | -0.52 | 0.47 | 0.65 | 0.71 |
| IGSF6 | 0.42 | -0.46 | 0.79 | -0.99 | -0.74 | -0.35 |
| CD1C | 0.42 | 0.32 | -0.50 | 0.63 | 0.38 | 0.19 |
| DAPP1 | 0.41 | -0.10 | 0.59 | -0.56 | -0.62 | -0.23 |
| LTA4H | 0.41 | -0.42 | 0.89 | -1.15 | -1.34 | -0.14 |
| MS4A7 | 0.40 | -0.40 | 0.59 | -1.40 | -1.23 | -0.09 |
| ABI3 | 0.40 | -0.55 | 0.71 | -0.87 | -0.68 | 0.16 |
| HOXB6 | 0.40 | -0.43 | 0.80 | -0.83 | -1.37 | -0.22 |
| CPVL | 0.39 | -0.23 | 0.61 | -1.08 | -1.15 | -0.01 |
| MRC1 | 0.37 | -0.60 | 0.56 | -0.81 | -1.26 | 0.72 |
| APOBR | 0.37 | -0.58 | 0.65 | -0.65 | -1.12 | -0.61 |
| PTPRJ | 0.36 | -0.73 | 0.63 | -0.94 | -1.24 | -0.69 |
| NFAM1 | 0.36 | -0.78 | 0.71 | -0.55 | -0.57 | 0.27 |
| NDNF | 0.34 | 0.00 | -0.82 | 0.87 | -0.42 | 0.34 |
| JPH4 | 0.33 | -0.88 | 0.67 | -0.95 | -0.46 | 0.21 |
| NLRC4 | 0.32 | -0.58 | 0.94 | -0.37 | -0.46 | 0.37 |
| FFAR2 | 0.30 | -0.59 | 0.32 | 0.60 | 0.34 | 0.53 |
| SCIMP | 0.29 | -0.16 | 0.84 | -0.38 | 0.33 | 0.43 |
| C15orf48 | 0.29 | 0.04 | -0.31 | 0.82 | 0.27 | -0.59 |
| TGFBR1 | 0.27 | -0.74 | 0.59 | -1.57 | -1.90 | -0.76 |
| H3C1 | 0.25 | -1.33 | -1.61 | 0.96 | 0.41 | -2.96 |
| HLA-DOA | 0.23 | -1.27 | 0.69 | -0.73 | -0.90 | 0.03 |
| GZMH | 0.22 | 0.81 | -0.38 | 1.15 | 1.42 | 1.05 |
| ENPP5 | 0.21 | -0.22 | -0.78 | 0.66 | -0.58 | 0.06 |
| MERTK | 0.21 | -0.80 | 0.61 | -1.37 | -1.81 | -0.88 |
| SLC25A19 | 0.21 | -0.66 | 0.71 | -1.88 | -1.51 | -0.29 |
| SLC16A3 | 0.20 | -0.51 | 0.60 | -1.03 | -1.14 | -0.74 |
| FCER1A | 0.20 | -0.25 | -0.99 | 1.04 | 0.14 | -0.13 |
| MARCO | 0.18 | -0.80 | 0.81 | -1.12 | -0.96 | 0.38 |
| LAIR1 | 0.18 | -0.65 | 0.61 | -1.02 | -1.41 | 0.17 |
| CLEC12A | 0.18 | -0.33 | 0.67 | -1.29 | -0.99 | -0.15 |
| CLEC10A | 0.18 | -0.63 | -0.51 | 0.39 | 0.01 | 0.87 |
| CFP | 0.18 | -0.14 | -0.27 | 0.62 | 0.47 | 1.05 |
| IL7R | 0.15 | 0.88 | -1.10 | 0.02 | -0.61 | -1.36 |
| RNASE3 | 0.12 | -0.20 | 0.11 | 0.66 | 0.20 | 0.66 |
| TLR2 | 0.12 | -0.05 | 0.69 | -1.20 | -1.63 | -0.94 |
| GPBAR1 | 0.10 | -0.91 | 0.35 | -0.03 | -0.47 | 0.92 |
| S1PR4 | 0.08 | 0.36 | 0.05 | 0.66 | 0.11 | 0.43 |
| IGFBP1 | 0.06 | -0.43 | 1.38 | -1.88 | -4.21 | -4.76 |
| PLA2G2D | 0.04 | -0.42 | 0.63 | -1.60 | -1.91 | -1.71 |
| SV2B | 0.02 | -1.17 | -0.27 | 0.04 | -1.56 | 0.90 |
| RETN | -0.00 | 0.68 | -0.75 | 0.27 | -0.60 | 0.61 |
| PKP1 | -0.03 | -0.88 | 0.81 | -1.11 | -0.89 | -0.44 |
| PRF1 | -0.05 | 0.39 | -1.10 | 0.89 | 0.97 | 1.22 |
| PRAM1 | -0.07 | -0.47 | 0.59 | -0.89 | -1.64 | -0.15 |
| MMP9 | -0.08 | -0.28 | -0.13 | -0.29 | -0.21 | 0.77 |
| NKG7 | -0.08 | 0.37 | -1.33 | 0.97 | 0.79 | 0.45 |
| KCNJ2 | -0.11 | -0.66 | 0.79 | -0.87 | -1.35 | -0.03 |
| FCMR | -0.24 | 0.73 | -2.12 | -0.47 | -1.04 | -0.97 |
| MATK | -0.30 | -0.45 | -0.28 | 0.62 | 0.41 | -0.28 |
| VWF | -0.38 | -2.17 | 0.65 | -1.39 | -1.43 | -0.93 |
| GZMB | -0.72 | -0.26 | -1.20 | 0.79 | 0.67 | 0.62 |
| CAVIN2 | -1.19 | -1.90 | -0.89 | -0.75 | -1.27 | 0.89 |
| IL33 | -1.22 | -1.59 | -0.16 | -0.59 | -0.58 | 0.90 |
| STC1 | -1.24 | -3.71 | -0.14 | -0.87 | -1.05 | 1.61 |
| XPNPEP2 | -1.79 | -2.10 | -2.30 | 0.89 | -0.34 | 0.73 |
| MFAP5 | -2.16 | -2.24 | -2.26 | 0.54 | 0.32 | 0.73 |
| PROK1 | -2.71 | -2.47 | -3.79 | 0.39 | 1.48 | 1.41 |
| GDF10 | -3.04 | -2.51 | -3.31 | 0.66 | -0.86 | -0.70 |
| PI16 | -3.42 | -3.60 | -2.63 | 1.23 | 0.32 | 1.20 |
| CD300LG | -4.62 | -1.79 | -4.18 | 0.88 | 0.33 | -2.81 |
| CMA1 | -6.07 | -4.85 | -2.66 | -3.10 | -3.81 | 0.65 |

**Supplementary Table 2: Genes where Fold Change > 1.5 between RA and OA under each condition**

| **Intact** | **Liberase** | **LibFlavo** | **SubA** |
| --- | --- | --- | --- |
| EGR1 | COMP | COL1A1 | FOSB |
| COL1A1 | SPP1 | CCN1 | SOCS3 |
| COL3A1 | HBA2 | BHLHE40 | CD248 |
| MMP3 | G0S2 | SPP1 | SPP1 |
| CCN1 | FABP4 | HBA2 | IGFBP6 |
| SPARC | HBB | ID1 | FSTL1 |
| PPP1R15A | HLA-B | FABP4 | HBA2 |
| KLF6 | MTRNR2L1 | HBB | G0S2 |
| SPP1 | CLEC14A | HLA-B | THBD |
| HBA2 | CD93 | ACKR1 | FABP4 |
| RGS1 | C3 | MTRNR2L1 | HBB |
| MMP1 | FABP5 | TAGLN | HLA-B |
| FABP4 | ASPN | CLEC14A | CCL2 |
| HBB | MARCKSL1 | CD93 | THY1 |
| HLA-B | CKB | C3 | CDKN1A |
| RGS2 | ITM2A | IGFBP3 | MTRNR2L1 |
| CCL2 | APLNR | MXRA5 | ADAMTS1 |
| CCR1 | CXCL14 | CILP | C3 |
| THY1 | NKG7 | HSPB6 | IGFBP3 |
| CDKN1A | CXCL2 | ASPN | RAMP2 |
| MTRNR2L1 | HSPA6 | MARCKSL1 | ASPN |
| SRGN | SOX18 | ITM2A | MARCKSL1 |
| CLEC14A | TNFAIP3 | APLNR | CKB |
| PHLDA1 | MFAP5 | CXCL14 | MMP9 |
| LRRC15 | DPT | NKG7 | ITM2A |
| ADAMTS1 | APOD | CXCL2 | APLNR |
| CD93 | TM4SF1 | HSPA6 | CXCL14 |
| IGFBP3 | FHL1 | SPRY1 | RGS16 |
| APOBR | CD34 | SOX18 | PIM1 |
| PNP | CD300E | MFAP5 | HSPG2 |
| TCIM | PLIN4 | CXCL8 | NKG7 |
| S1PR1 | CRABP2 | PODN | CXCL1 |
| FIBIN | SLC2A3 | APOD | CXCL2 |
| MARCKSL1 | FAM180B | MCAM | FOSL2 |
| APLNR | HMGA1 | TM4SF1 | VCAN |
| ACTR2 | FBLN2 | SLPI | SPRY1 |
| EVI2B | CLDN5 | FHL1 | AOC3 |
| TNFAIP6 | RARRES2 | CD34 | EHD2 |
| NKG7 | PLVAP | CD300E | MFAP5 |
| CXCL1 | HBA1 | PLIN4 | PODN |
| CXCL2 | CXCL10 | BCAM | APOD |
| HSPA6 | VEGFA | CRABP2 | BTN3A1 |
| SPRY1 | CXCL9 | SLC2A3 | MCAM |
| AOC3 | GBP1 | CCR5 | DBN1 |
| TNFAIP3 | TINAGL1 | FAM180B | CSRNP1 |
| CXCL8 | NES | FBLN2 | SLPI |
| APOD | LIMD2 | CLDN5 | CD34 |
| MCAM | GPD1 | RARRES2 | PKDCC |
| ICAM1 | NR4A2 | POSTN | PLIN4 |
| SAMD9L | CXCL3 | CLEC10A | NR4A1 |
| DOK3 | PLIN1 | CH25H | CRABP2 |
| SERPINE1 | EGFL7 | PLVAP | SLC2A3 |
| TNFRSF12A | LOXL1 | HBA1 | FAM180B |
| CD300E | ESAM | CXCL10 | HOXD4 |
| PLIN4 | GJA4 | THBS1 | FBLN2 |
| NR4A1 | PI16 | FBLN1 | CLDN5 |
| RANBP6 | FGFBP2 | CXCL9 | RARRES2 |
| APOL6 | LCP1 | TINAGL1 | CLEC10A |
| SLC2A3 | RFLNB | SNCG | FBN1 |
| CCR5 | SOX17 | NES | TAP1 |
| FASN | HCST | HRCT1 | NAMPT |
| POSTN | FCN1 | NR1D1 | CDC42EP1 |
| FHOD1 | TMC8 | GPD1 | HBA1 |
| CH25H | GBP4 | AMOTL2 | CXCL10 |
| WARS1 | CAMK2N1 | IGFBP7 | VEGFA |
| SLC20A1 | NBEAL2 | CXCL3 | THBS1 |
| EGR3 | NPR1 | PLIN1 | FBLN1 |
| PLVAP | PIM2 | EGFL7 | CXCL9 |
| TAP1 | LRRC32 | ESAM | LYVE1 |
| NAMPT | ROBO4 | CORO6 | GBP1 |
| CXCL10 | CTSW | GJA4 | NES |
| VEGFA | ADAM33 | PI16 | CNN3 |
| THBS1 | CD69 | ACVRL1 | LIMD2 |
| MSC | GPIHBP1 | LCP1 | HRCT1 |
| CXCL9 | IL2RG | RFLNB | ITM2C |
| RBM12 | ITGAX | SOX17 | GPD1 |
| THBS2 | RAC2 | PLK2 | AMOTL2 |
| GBP1 | ADIPOQ | FCN1 | TNFSF10 |
| TINAGL1 | RASAL3 | GBP4 | SYNPO |
| TLR2 | HES4 | CAMK2N1 | LOX |
| NES | ADAM8 | EDN1 | DUSP2 |
| LTBP2 | PRF1 | NBEAL2 | EGFL7 |
| GASK1B | TMEM88 | NPR1 | JAG1 |
| GPD1 | TBC1D10C | LRRC32 | SEMA3G |
| IGFBP7 | CDH5 | ROBO4 | HIC1 |
| NR4A2 | C1orf115 | ADCY4 | LOXL1 |
| CXCL3 | ARHGEF15 | CIITA | GJA4 |
| PLIN1 | ACTN1 | CTSW | HOXD9 |
| EPAS1 | GZMB | ADAM33 | PI16 |
| COL12A1 | SFRP4 | CD69 | FGFBP2 |
| DUSP2 | CTHRC1 | GPIHBP1 | APOL3 |
| CAPZA1 | KIAA0040 | MAPK13 | ACVRL1 |
| FCGR1A | CCL21 | IL2RG | CAVIN2 |
| ESAM | MYOC | ITGAX | SOX17 |
| KPNA2 | COL5A3 | MAPK11 | PLK2 |
| GJA4 | OLFML3 | SPHK1 | MFAP2 |
| PI16 | SPN | RAC2 | C1QTNF3 |
| GPR183 | CD79B | PALM | FCN1 |
| HSPH1 | IGLL5 | ADIPOQ | MSX1 |
| ACVRL1 | GPR84 | RASAL3 | GALNT15 |
| NFKBIE | NRARP | FLNC | GBP4 |
| LCP1 | BCL6B | HES4 | ADAMTS5 |
| RFLNB | GBP5 | ADAM8 | SLC44A2 |
| COL4A2 | ACE | PRF1 | CAMK2N1 |
| NME1 | ANGPTL7 | TMEM88 | HLX |
| PPRC1 | CCL14 | CD83 | TGM2 |
| LACC1 | BIRC3 | CDH5 | SERPINA1 |
| PLK2 | CILP2 | NOTCH3 | CASKIN2 |
| FCN1 | MAFF | C1orf115 | CLSTN3 |
| COL4A1 | GZMH | ARHGEF15 | NBEAL2 |
| TGFBR1 | IL32 | ACTN1 | NPR1 |
| TMC8 | MZB1 | VWF | OLFML1 |
| SIGLEC10 | HSPB7 | GZMB | NFIL3 |
| PICALM | JCHAIN | CTHRC1 | NRN1 |
| ERAP1 | BTNL9 | CCL8 | CTSW |
| RESF1 | CD1C | MYOC | ADAM33 |
| GBP4 | FAM167B | MMRN2 | CD69 |
| NACC1 | ADH1B | COL5A3 | CD7 |
| GBP3 | DACT2 | SPN | CD209 |
| CPXM1 | SPON2 | COL15A1 | GPIHBP1 |
| TGM2 | MEOX1 | EPHB4 | IL2RG |
| CASKIN2 | CX3CL1 | GPR84 | VENTX |
| ORAI2 | CLEC5A | NRARP | ITGAX |
| EDEM1 | MAP1LC3C | BCL6B | NOTCH1 |
| PIM2 | ADRA2A | OLFML2A | RAC2 |
| SAMD9 | SLC5A3 | GBP5 | PALM |
| ROBO4 | KCNA3 | ACE | ADIPOQ |
| ADCY4 | SHANK3 | CCL14 | FLNC |
| KBTBD2 | CD3E | BIRC3 | ADAM8 |
| CD69 | S100B | LAG3 | CLIC3 |
| MAPK13 | CD79A | LIMS2 | SLC25A22 |
| IL2RG | CD3D | MAFF | PRF1 |
| ITGAX | ACTA2 | FNDC1 | TMEM88 |
| MAPK11 | IL7R | DYNLT4 | TBC1D10C |
| SPHK1 | PILRB | GZMH | ITGA7 |
| NOTCH1 | INHBB | HAPLN3 | NOTCH3 |
| TES | CXCR3 | IL32 | ARHGEF15 |
| PVR | LIPE | TIE1 | ACTN1 |
| RAC2 | CIDEC | HSPB7 | SNAI1 |
| ADIPOQ | HSPA12B | MEST | SERTAD4 |
| TRIB2 | CCR2 | IL6 | GZMB |
| ADAM8 | HCAR2 | BTNL9 | SFRP4 |
| SLC25A22 | PKN3 | CD1C | CTHRC1 |
| LAT2 | ATF3 | FAM167B | CCL8 |
| PTPRC | GZMA | IL27RA | MYOC |
| SERPINB9 | CCL19 | ADH1B | COL5A3 |
| PRF1 | F2R | SPON2 | SPN |
| ZWINT | SLAMF8 | MEOX1 | COL15A1 |
| CD83 | THRSP | TFRC | CD79B |
| MDM2 | HEY1 | ITGB4 | TBKBP1 |
| TBC1D10C | SOX15 | CX3CL1 | GJB6 |
| ACSL1 | ARAP3 | PODXL | NDST1 |
| NRP2 | FAS | CLEC5A | IGLL5 |
| CDH5 | TNFRSF4 | MAP1LC3C | GPR84 |
| SDE2 | LPL | DUSP5 | BCL6B |
| NOTCH3 | PPP1R14A | ADRA2A | OLFML2A |
| ARHGEF15 | RASIP1 | KCNA3 | ACAP1 |
| ACTN1 | JAG2 | FAM110D | GBP5 |
| STK17B | AIF1L | SHANK3 | ACE |
| STAT1 | AQP7 | LGI4 | ANGPTL7 |
| VWF | DLL4 | CD3E | GDPD3 |
| GZMB | SLAMF7 | S100B | AEN |
| CTHRC1 | PTPN7 | IL1B | BIRC3 |
| CCL8 | DUSP4 | CD3D | LAG3 |
| LCP2 | PEAR1 | ACTA2 | LIMS2 |
| KIAA0040 | RRAD | PILRB | MAFF |
| MTHFD2 | BAMBI | INHBB | FNDC1 |
| COL5A3 | TCAP | GATA2 | PTHLH |
| SLC2A6 | TNFAIP8L1 | CXCR3 | GZMH |
| ATP6V1A | CHAD | NID1 | HAPLN3 |
| SPN | TMEM204 | CD2 | EFNA1 |
| COL15A1 | FOXS1 | LIPE | IL32 |
| MARS2 | TBX2 | CIDEC | MZB1 |
| MMP13 | WNT11 | HSPA12B | HSPB7 |
| CD79B | SOX7 | HLA-DOA | JCHAIN |
| PENK | PCDH1 | CCR2 | TGFB3 |
| GPR65 | SPRY4 | HCAR2 | MEST |
| IGLL5 | TNFRSF18 | PKN3 | IL6 |
| GPR84 | C19orf73 | ATF3 | JAK3 |
| NRARP | IL33 | GZMA | BTNL9 |
| BCL6B | ITGA11 | F2R | SOCS2 |
| ACAP1 | NECTIN4 | NOS3 | TRAF4 |
| GBP5 | C11orf21 | GSDMB | CD1C |
| ZNF217 | LOXL2 | KCTD15 | CX3CR1 |
| MICALL2 | AKAP12 | SLAMF8 | ADH1B |
| AEN | ADRA2C | SH2D3C | DACT2 |
| BIRC3 | WNT10B | THRSP | ETS1 |
| MAFF | GNLY | HEY1 | MEOX1 |
| FNDC1 | LDLR | ARAP3 | AKNA |
| GZMH | ITGAL | NFKBID | CX3CL1 |
| OTULIN | KANK3 | AVPR2 | SIT1 |
| HAPLN3 | XPNPEP2 | TNFRSF4 | PODXL |
| FZD5 | S100A12 | GPT | CLEC5A |
| IL32 | MATK | SUSD2 | PLAT |
| MZB1 | KCNIP2 | AKR1C3 | MAP1LC3C |
| HMGCS1 | ADAMTS4 | TSPAN33 | DUSP5 |
| TIE1 | SPOCK2 | PPP1R14A | ADRA2A |
| RRM2B | MATN2 | RASIP1 | SLC5A3 |
| HSPB7 | SEMA6B | PTGIS | ATP2A3 |
| JCHAIN | GPRC5B | JAG2 | PTGS2 |
| IL6 | HIP1R | AIF1L | KCNA3 |
| MSMO1 | GDF10 | AQP7 | PNMA2 |
| JAK3 | C2CD4B | CYP26B1 | PLPP1 |
| BTNL9 | KDR | DLL4 | LGI4 |
| FICD | IRX6 | SLAMF7 | CDC20 |
| CD1C | RBP7 | PTPN7 | OSM |
| FAM167B | TPBGL | DUSP4 | CD3E |
| CCDC88A | RNF207 | GPR4 | S100B |
| ADH1B | GPR68 | DIPK2B | CD79A |
| LBR | CD8A | SLFN13 | IL1B |
| ETS1 | CST7 | PEAR1 | TMEM47 |
| MEOX1 | SCRN1 | RRAD | CD3D |
| TFRC | SYTL1 | BAMBI | ACTA2 |
| SELL | DPP4 | SIK1B | IL7R |
| HMGCR | CHRDL1 | TNFAIP8L1 | PILRB |
| SIT1 | CD48 | KRT18 | INHBB |
| GNB4 | LOXL4 | TMEM204 | ADM5 |
| CDKN2B | APLN | FOXS1 | NXPH3 |
| PODXL | SLC38A5 | TBX2 | SEMA6C |
| CHST15 | CALHM5 | WNT11 | CXCR3 |
| CLEC5A | SEMA3F | SOX7 | NID1 |
| DUSP5 | TBX3 | PCDH1 | BMP2 |
| ZC3HAV1 | GALNT16 | SPRY4 | LIPE |
| PTGS2 | KCNA1 | IL33 | VGLL2 |
| KCNA3 | BCL2A1 | ITGA11 | LIF |
| ARHGAP18 | CD300LG | C11orf21 | S1PR2 |
| TTPAL | TRAF3IP3 | LOXL2 | CIDEC |
| SMC4 | CCDC163 | AKAP12 | HSPA12B |
| SLC25A24 | PNMT | ADRA2C | CCR2 |
| ST14 | ADH1C | WNT10B | HCAR2 |
| SHANK3 | S1PR4 | PCDHB8 | PKN3 |
| CDC20 | FOSL1 | LDLR | ATF3 |
| OSM | MMP15 | ITGAL | GZMA |
| SPRED1 | RNF125 | KANK3 | ITGB3 |
| CD3E | CST6 | S100A12 | GABRE |
| CD79A | C1QTNF2 | MATK | CCL19 |
| CKAP2 | ZAP70 | SPNS2 | PRDM8 |
| NPC1 | AQP9 | KCNIP2 | NOS3 |
| INHBA | ITGB7 | ADAMTS4 | ITIH5 |
| IL1B | ICAM3 | SPOCK2 | SLAMF8 |
| CD3D | MEIS3 | MATN2 | SH2D3C |
| ACTA2 | AVPR1A | TM4SF18 | CD24 |
| PPIL1 | ISG20 | SEMA6B | THRSP |
| IL7R | CD27 | GPRC5B | HEY1 |
| GAPT | CD1E | GDF10 | FAS |
| PILRB | CNN1 | C2CD4B | KCNA6 |
| SQLE | GZMK | KDR | TNFRSF4 |
| MICALL1 | CDK18 | ABLIM1 | EFNB2 |
| SGPL1 | FAM167A | IRX6 | SEPTIN1 |
| CEMIP | CCR7 | LMCD1 | MKI67 |
| CXCR3 | FNDC5 | CD8A | SCNN1D |
| NID1 | MDFI | KCNJ8 | RGS11 |
| BMP2 | ITGA4 | CST7 | NLRC5 |
| LIPE | EPHA2 | SAP25 | GJD3 |
| STARD4 | CD36 | PRRG3 | PPP1R14A |
| CIDEC | GPAM | SYTL1 | LRRC4 |
| PIK3AP1 |  | DPP4 | PTGIS |
| HSPA12B |  | NUAK1 | JAG2 |
| HLA-DOA |  | DIPK1B | AIF1L |
| CCR2 |  | CHRDL1 | AQP7 |
| CKS2 |  | APLN | DLL4 |
| HCAR2 |  | FUT7 | BAHCC1 |
| ATF3 |  | RAC3 | SLAMF7 |
| GZMA |  | CALHM5 | PTPN7 |
| C3orf80 |  | STC1 | DUSP4 |
| PIK3CG |  | SEMA3F | GPR4 |
| CCL19 |  | PTX3 | BAMBI |
| F2R |  | TBX3 | RETN |
| DSC2 |  | HID1 | SIK1B |
| NOD2 |  | TMEM132A | ZNF853 |
| NOS3 |  | BCL2A1 | TNFAIP8L1 |
| GSDMB |  | CD300LG | CHAD |
| TUBA1C |  | MYCT1 | FOXS1 |
| UNC5B |  | CCDC163 | WNT11 |
| SLAMF8 |  | FST | SOX7 |
| SUCNR1 |  | CARD10 | SPRY4 |
| SH2D3C |  | PTPRB | TNFRSF18 |
| TMEM87B |  | ADH1C | FMO2 |
| CD24 |  | EXOC3L1 | IL33 |
| NBN |  | SULT1C4 | C11orf21 |
| THRSP |  | MMP15 | PLXNB1 |
| HEY1 |  | MGLL | LOXL2 |
| ARAP3 |  | JAM2 | AKAP12 |
| NFKBID |  | NEURL1B | ADRA2C |
| FCGR1B |  | C1QTNF2 | NKAPL |
| FAS |  | SCN4B | WNT10B |
| AVPR2 |  | CA2 | GNLY |
| FYB1 |  | ZAP70 | TUBA4A |
| TNFRSF4 |  | AQP9 | SMOC1 |
| SEMA4A |  | LHX6 | DCUN1D3 |
| MKI67 |  | ECSCR | LDLR |
| DUSP10 |  | POMC | ITGAL |
| NLRC5 |  | MEIS3 | CCZ1B |
| FPR1 |  | AVPR1A | XPNPEP2 |
| RASIP1 |  | CYTL1 | SYNM |
| LRRC4 |  | H3C1 | S100A12 |
| JAG2 |  | CD1E | KCNIP2 |
| UBE2C |  | CNN1 | ADAMTS4 |
| AQP7 |  | GZMK | SPOCK2 |
| PSME4 |  | HEYL | MATN2 |
| CYP26B1 |  | LRRC70 | PAFAH1B3 |
| SCYL2 |  | FNDC5 | SEMA6B |
| PODNL1 |  | MDFI | CDT1 |
| SLAMF7 |  | ITGA4 | GPRC5B |
| PTPN7 |  | EPHA2 | AOX1 |
| DUSP4 |  | CD36 | HIP1R |
| GPR4 |  | SLCO2A1 | GDF10 |
| NABP1 |  | MAOA | C2CD4B |
| ZBED4 |  | PACSIN3 | EPHB3 |
| DIPK2B |  | SEPTIN5 | ABLIM1 |
| CYRIB |  |  | IRX6 |
| JCAD |  |  | RNF207 |
| SIK1B |  |  | TMEM121 |
| CCNYL1 |  |  | CCIN |
| WDFY1 |  |  | LMCD1 |
| SNX10 |  |  | GPR68 |
| GPRIN3 |  |  | CD8A |
| TNFAIP8L1 |  |  | CA12 |
| BEX2 |  |  | CST7 |
| CBL |  |  | CRIM1 |
| FOXS1 |  |  | SCRN1 |
| DSE |  |  | HOXC6 |
| HEATR3 |  |  | PRRG3 |
| TBX2 |  |  | SYTL1 |
| MXD1 |  |  | DPP4 |
| SOX7 |  |  | DIPK1B |
| PCDH1 |  |  | CHRDL1 |
| IKZF1 |  |  | GP1BA |
| SPRY4 |  |  | CD48 |
| NECTIN4 |  |  | LOXL4 |
| C11orf21 |  |  | APLN |
| LOXL2 |  |  | SLC38A5 |
| GNLY |  |  | PIK3C2B |
| SGTB |  |  | RAC3 |
| CLEC4E |  |  | CALHM5 |
| CDH11 |  |  | STC1 |
| LDLR |  |  | TK1 |
| RGS3 |  |  | PLD6 |
| ITGAL |  |  | CD1D |
| CCZ1B |  |  | ARID5B |
| TNFRSF10D |  |  | TBX3 |
| S100A12 |  |  | C21orf91 |
| SPNS2 |  |  | FGF7 |
| KCNIP2 |  |  | DMRT2 |
| ADAMTS4 |  |  | RASSF5 |
| SPOCK2 |  |  | GALNT16 |
| MATN2 |  |  | SH2D2A |
| STK17A |  |  | KIF20A |
| TM4SF18 |  |  | KCNA1 |
| SEMA6B |  |  | HEY2 |
| MAPK12 |  |  | BCL2A1 |
| COL8A1 |  |  | CD300LG |
| C2CD4B |  |  | ODF3L1 |
| BAZ1A |  |  | TRAF3IP3 |
| TASL |  |  | CCDC163 |
| SERPINE2 |  |  | FST |
| KDR |  |  | ADH1C |
| EPHB3 |  |  | S1PR4 |
| RNF207 |  |  | SULT1C4 |
| SLC16A6 |  |  | TOP2A |
| SLC19A3 |  |  | FOSL1 |
| GPR68 |  |  | FZD6 |
| CD8A |  |  | CST6 |
| CRIM1 |  |  | CALCRL |
| SYTL1 |  |  | C1QTNF2 |
| NUAK1 |  |  | SCN4B |
| CD48 |  |  | SPAG4 |
| APLN |  |  | TSPAN11 |
| SLC38A5 |  |  | ZAP70 |
| TLR1 |  |  | ITGB7 |
| RAC3 |  |  | HYAL3 |
| TK1 |  |  | FBLIM1 |
| SEMA3F |  |  | ICAM3 |
| PTX3 |  |  | DGKE |
| CD1D |  |  | MEIS3 |
| TBX3 |  |  | GPR17 |
| FGF7 |  |  | AVPR1A |
| DOCK8 |  |  | ISG20 |
| PTPRJ |  |  | DTX4 |
| TMEM132A |  |  | CD27 |
| DLX5 |  |  | CD1E |
| SH2D2A |  |  | STXBP1 |
| KIF20A |  |  | CNN1 |
| PLK1 |  |  | COL27A1 |
| EGR2 |  |  | GZMK |
| SIX4 |  |  | RAI14 |
| BCL2A1 |  |  | CRISPLD2 |
| CD300LG |  |  | HEYL |
| MYCT1 |  |  | BIRC5 |
| EML4 |  |  | LRRC70 |
| ADGRE2 |  |  | CCR7 |
| RUNX3 |  |  | VN1R1 |
| TRAF3IP3 |  |  | FNDC5 |
| CTPS1 |  |  | SPRED3 |
| CCDC163 |  |  | ITGA4 |
| UHRF1BP1 |  |  | ADAMTSL5 |
| BMP2K |  |  | CD36 |
| EXOC3L1 |  |  | MPDZ |
| PROB1 |  |  | SEPTIN4 |
| FAM166B |  |  | MT1H |
| TOP2A |  |  | DHRS13 |
| FOSL1 |  |  | PACSIN3 |
| MMP15 |  |  | TLE2 |
| TET3 |  |  | RNASEH2A |
| ZNF267 |  |  | EBF3 |
| KLHL5 |  |  |  |
| RNF125 |  |  |  |
| CST6 |  |  |  |
| SEZ6L2 |  |  |  |
| NEURL1B |  |  |  |
| CDC6 |  |  |  |
| SPAG4 |  |  |  |
| CA2 |  |  |  |
| TYMS |  |  |  |
| ZAP70 |  |  |  |
| AQP9 |  |  |  |
| JAK2 |  |  |  |
| LHX6 |  |  |  |
| ITGB7 |  |  |  |
| ICAM3 |  |  |  |
| DMKN |  |  |  |
| CXCL6 |  |  |  |
| AVPR1A |  |  |  |
| PRAG1 |  |  |  |
| CD1E |  |  |  |
| CGAS |  |  |  |
| SPAG5 |  |  |  |
| PHF6 |  |  |  |
| PRKACB |  |  |  |
| ZNF697 |  |  |  |
| GZMK |  |  |  |
| ADRB1 |  |  |  |
| ESPL1 |  |  |  |
| CDK18 |  |  |  |
| HEYL |  |  |  |
| RGS4 |  |  |  |
| BIRC5 |  |  |  |
| SPRED3 |  |  |  |
| MDFI |  |  |  |
| ITGA4 |  |  |  |
| SHLD3 |  |  |  |
| EPHA2 |  |  |  |
| CD36 |  |  |  |
| DAPP1 |  |  |  |
| SEPTIN4 |  |  |  |
| SRD5A1 |  |  |  |
| SHTN1 |  |  |  |
| PRKX |  |  |  |
| URB2 |  |  |  |
| ENTPD7 |  |  |  |
| SLC46A2 |  |  |  |
| GPAM |  |  |  |
| TRAF3 |  |  |  |
| RPP40 |  |  |  |
| IFNE |  |  |  |

**Supplementary Figure 1: FastQC Sequence Counts**


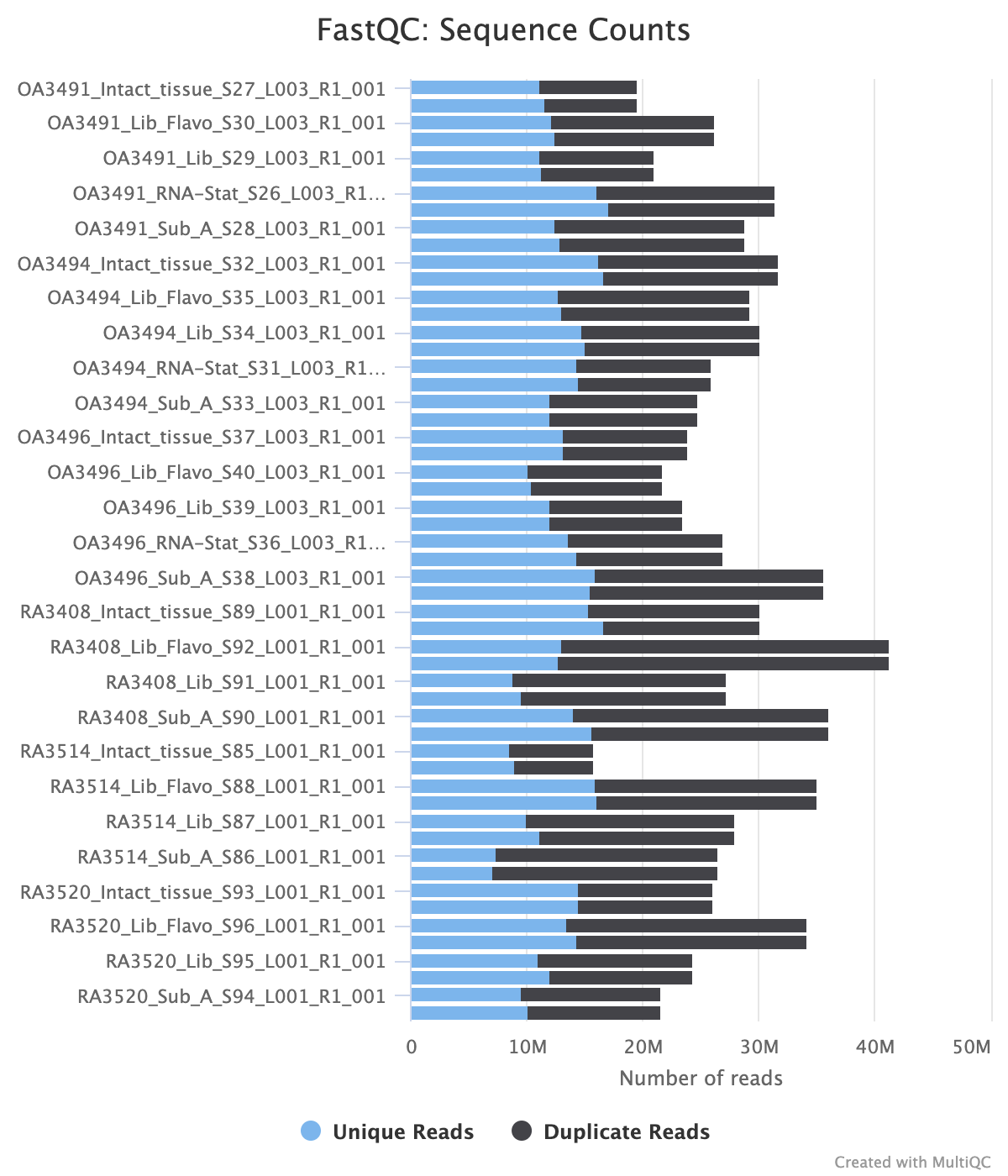


**Supplementary Figure 2: STAR Alignment Scores**


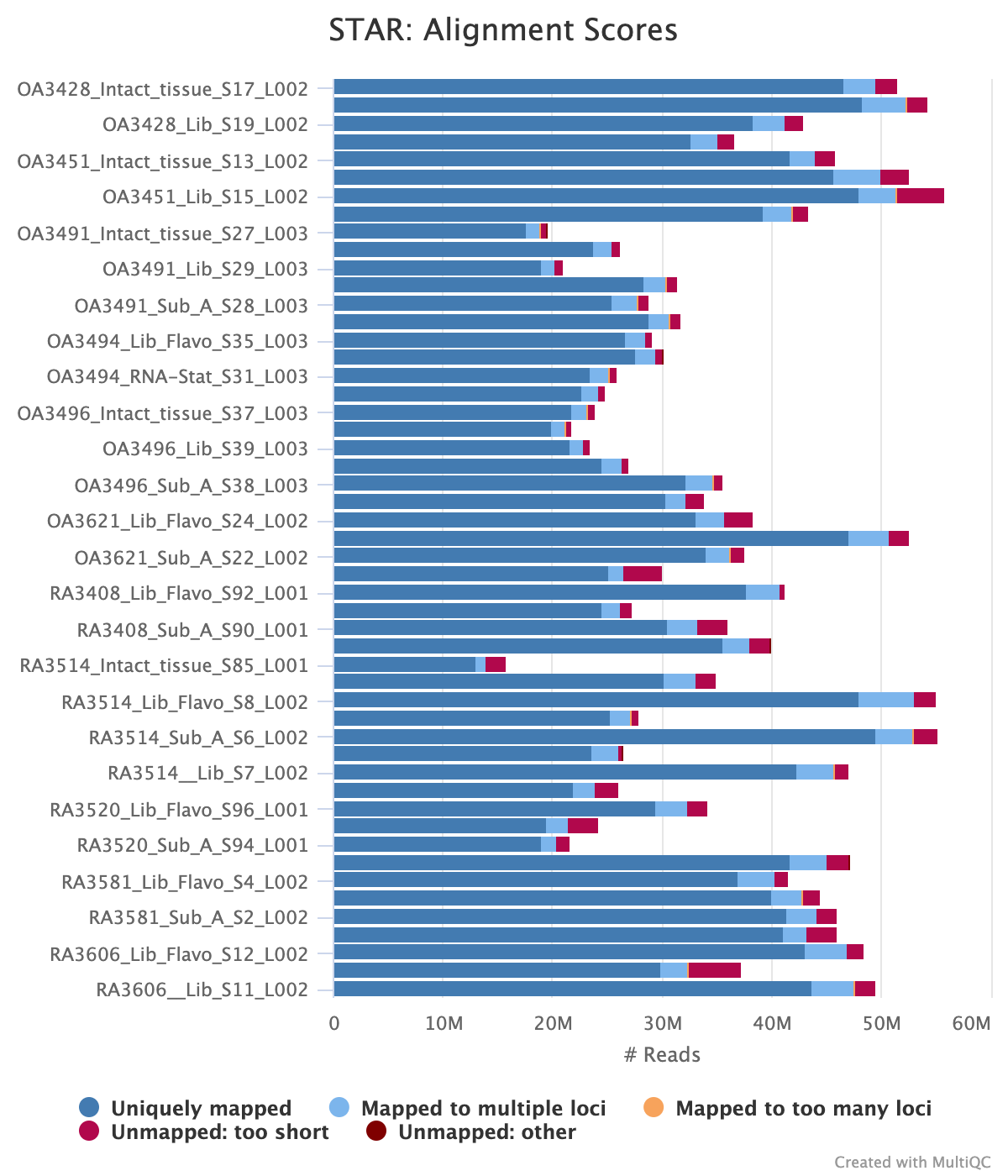


**Supplementary Figure 3: HTseq Count Assignments**


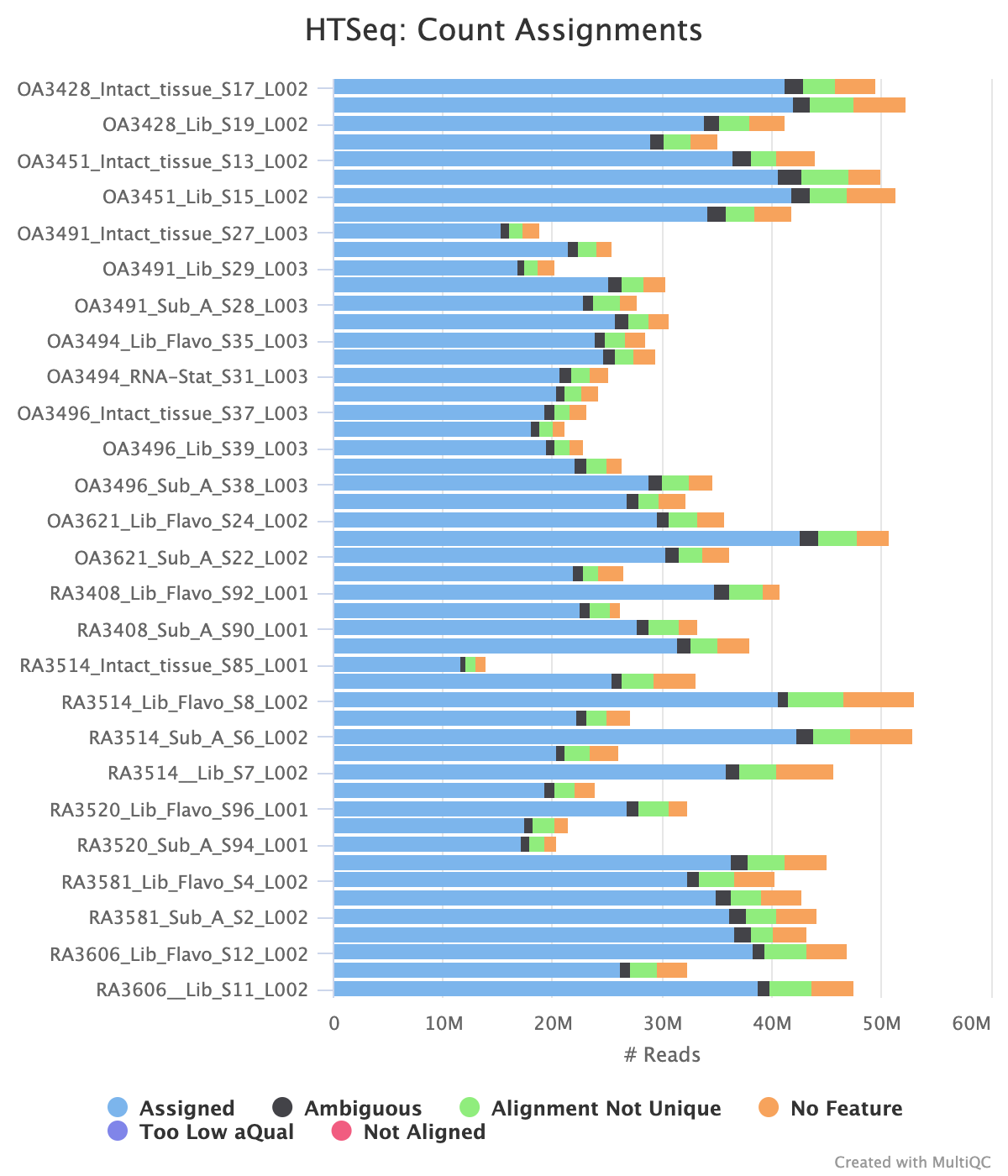


**Supplementary Figure 4: Mitochondrial and rRNA Reads**


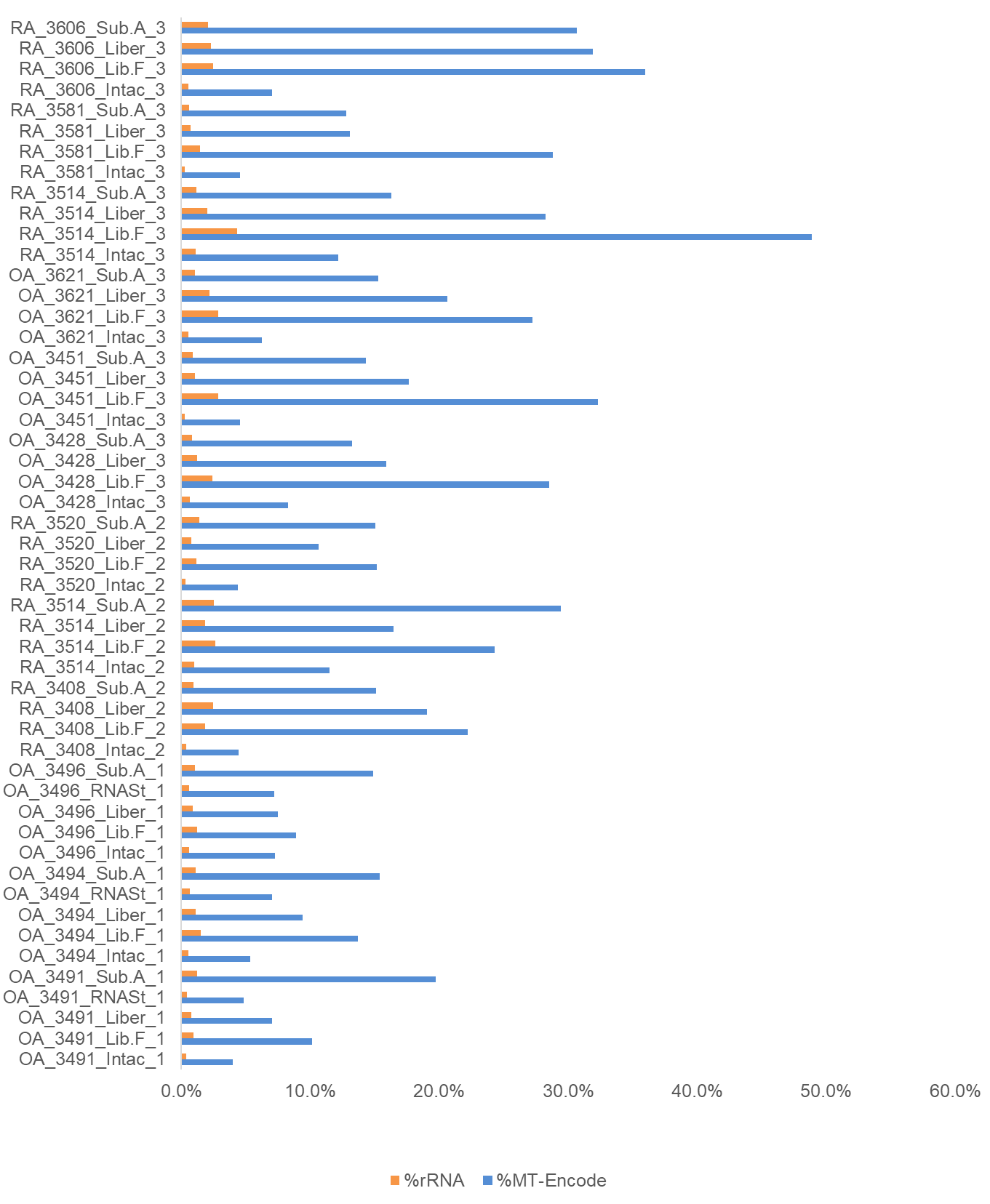

Supplement: Supplementary file 1 [file Data_Sheet_1.docx]
